# Self-healable electroluminescent devices

**DOI:** 10.1038/s41377-018-0096-8

**Published:** 2018-12-05

**Authors:** Guojin Liang, Zhuoxin Liu, Funian Mo, Zijie Tang, Hongfei Li, Zifeng Wang, Venkateshwarlu Sarangi, Abhijit Pramanick, Jun Fan, Chunyi Zhi

**Affiliations:** 1 Department of Materials Science and Engineering, City University of Hong Kong, 83 Tat Chee Avenue, Kowloon, China; 2 Shenzhen Research Institute, City University of Hong Kong, High-Tech Zone, Nanshan District Shenzhen, 518057 China

## Abstract

Electroluminescent (EL) devices have been extensively integrated into multi-functionalized electronic systems in the role of the vitally constituent light-emitting part. However, the lifetime and reliability of EL devices are often severely restricted by concomitant damage, especially when the strain exceeds the mechanical withstanding limit. We report a self-healable EL device by adopting a modified self-healable polyacrylic acid hydrogel as the electrode and a self-healable polyurethane as a phosphor host to realize the first omni-layer-healable light-emitting device. The physicochemical properties of each functionalized layer can be efficiently restored after experiencing substantial catastrophic damage. As a result, the luminescent performance of the self-healable EL devices is well recovered with a high healing efficiency (83.2% for 10 healing cycles at unfixed spots, and 57.7% for 20 healing cycles at a fixed spot). In addition, inter-device healing has also been developed to realize a conceptual “LEGO”-like assembly process at the device level for light-emitting devices. The design and realization of the self-healable EL devices may revive their performance and expand their lifetime even after undergoing a deadly cut. Our self-healable EL devices may serve as model systems for electroluminescent applications of the recently developed ionically conductive healable hydrogels and dielectric polymers.

## Electroluminescence: Self-healing electroluminescent devices

Inspired by the self-healing abilities of biological systems, scientists have developed an electroluminescent (EL) device that heals itself after being damaged, paving the way for a range of new applications. Although EL devices are used in a wide range of electronic applications, including backlights for the control panels, digital displays, and wearable electronics, they are prone to damage, which restricts their working life and reliability, limiting their applications. Now, Chunyi Zhi and colleagues from the City University of Hong Kong have developed the first self-healing EL device by using a modified self-healable polyacrylic acid hydrogel for electrodes and self-healable polyurethane as a phosphor host. Even after experiencing dozens of catastrophic damages, the physiochemical properties of the device can be restored and could be used in new applications, such as healable hydrogels and dielectric polymers.

## Introduction

Electroluminescent (EL) devices have been developed as indispensable modular elements in various commercially available electronic systems, such as the backlighting source in a car control panel^1,2^. Driven by diverse demand for versatile systems in daily life and integrated devices, EL devices have been applied in different fields, such as bioinspired soft robotics for visual disguise and artificial skin actuators^3,4^, flexible and stretchable electronics^5–10^, wearable electronics^11,12^, digital displays^8,10^, and sensors^3,13^. These exquisitely designed multifunctional EL devices benefit from the recent developments of transparent conductive materials, biological soft robotics, and optimized device configurations. For example, cephalopods-inspired camouflage and stretchable robots have been developed by changing the color of the light emitted from the integrated EL component^3,4^. Ag nanowires were exploited to render transparent electrodes for a self-deformable EL actuator for a volumetric content display^14^. Recently, extremely stretchable EL devices have been demonstrated in extensible ionically conductive hydrogels^7–9^. Coplanar EL devices and fiber-shaped EL devices have also been designed for wearable fabrics and optically communicating sensors, respectively^11–13^. These EL-based components in integrated electronic systems will satisfy extensive applicability with environmentally mechanical compliance. The primary concern in utilizing EL-integrated systems is the mechanical deformations and the concomitant damage. Despite the improved mechanical robustness of these devices, which have been realized by deploying robust functional materials and strain-minimizing device configuration, the degradation of device performance is not avoidable if the strain exceeds the withstanding limit. Maintaining and replacing a faulty component in a multifunctional integrated electronic system is either intractable or costly. Therefore, developing an effective strategy to avoid or minimize performance failure or decay of light-emitting devices under mechanical deformations has significance in terms of extending the lifetime of an EL device.

Inspired by the self-healing phenomena of natural biological systems, researchers have endowed artificial materials with similar healing properties to renovate and revive materials’ performances in situ. Healable materials have been designed and developed with either intrinsically healable features or extrinsically healable features, where damages can be autonomously repaired, or under external stimuli such as pH, light, electric, or magnetic fields^15–19^. After a healing process, these materials can restore their structure and physicochemical and mechanical properties, even in scenarios that involve substantial damage. Materials with different physicochemical properties, such as metal, ionic conductor, semiconductor, and dielectric polymer, have been demonstrated to have reversible healing abilities^20–25^. Despite the healing properties at the exquisitely designed material level, healable electronic devices that show potential for substantial improvement in device lifetime and reliability, have been rarely developed^18,19,26–28^. The majority of self-healing performances achieved for multilayered self-healable electronic devices were based on the self-healing properties of one constituent layer, whereas other functional layers were manually repaired by small patches or were not discussed in terms of self-healing^18,19,28^. Designing and realizing omni-layer-healable electronic devices are more challenging than designing and realizing single-layer-healable devices as each functionalized constituent layer is required to be self-healable in a complex configuration. Self-healable EL devices that involve different functionalized layers, such as electrodes, light-emitting layers, and other functionalized layers, have never been reported. The absence of a healable feature in light-emitting fields is attributed to the difficulties in designing and applying self-healable materials to each component of light-emitting devices, which should not only satisfy different requirements of each integrated functionalized layer but also restore their physicochemical properties from damage. Specifically, the design of electrodes for self-healing EL devices should consider several criteria and parameters^3,6,7^, such as visible-light transmittance and conductivity, as well as healability. Some criteria and parameters for the light-emission layer^7,9^, such as dielectric capacitance, mechanical strength, and healability, also exist. Due to the configuration complexity and synergy of different criteria of the physicochemical properties for each functionalized layer, designing and fabricating omni-layered self-healable EL devices are challenging.

By adopting modified self-healable polyacrylic acid (PAA) hydrogel that contains NaCl as an ionic electrode and self-healable polyurethane (PU) that contains ZnS particles as a phosphor composite layer, we demonstrate the first intrinsically highly self-healable EL devices with excellent self-healing capability. The as-fabricated self-healable EL devices were mechanically flexible, and the physicochemical properties of each individual healable layer can be well restored after experiencing catastrophic damage, such as several dozen incidences of cutting at fixed locations and unfixed locations. As a result, the luminescent performance of full devices can be effectively recovered, where the reliability and lifetime of EL devices are enhanced, compared with devices that are incapable of healing after undergoing mechanical damage. In addition to the healing of fracture damages within a single EL device, the inter-device healing has also been developed for the first time to enable an EL device-level assembly that is aimed at simplifying the complex and costly processes for repair and replacement of individual EL units in integrated electronic systems. Benefitting from inter-device healing, the conceptual “LEGO” assembly process at the device level for light-emitting devices is proposed and demonstrated.

## Results

### Working mechanism and materials formulation for self-healing EL devices

**Fig. 1:**
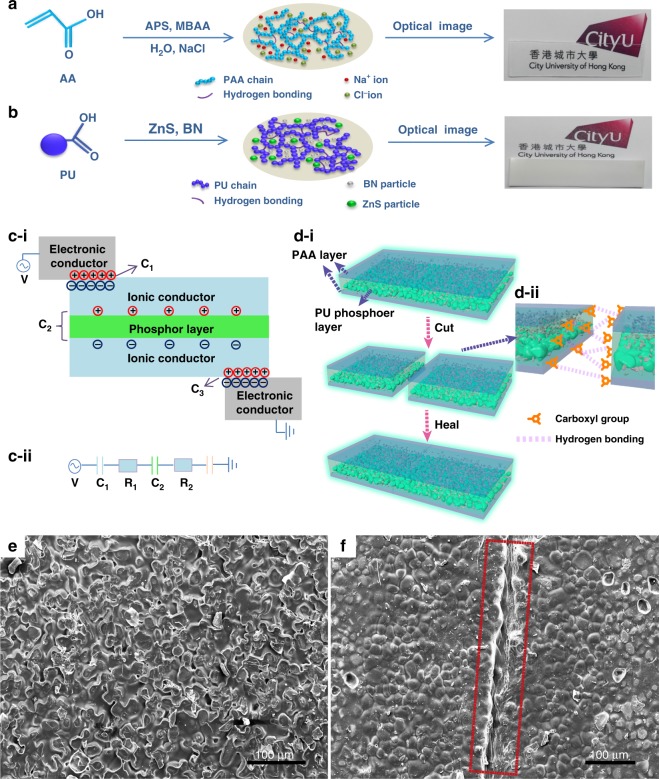
Fabrication process and self-healing mechanism. Synthesis process of the PAA/NaCl ionic conductor layer in (**a**) and the PU/ZnS/BN composite phosphor layer in (**b**), where the optical photographs of the as-obtained layers are shown. **c-i** Schematic principle and structure of the self-healable EL device. **c-ii** Equivalent circuit of an EL device, where C represents the corresponding capacitor and R represents the resistance of the ionic conductor. **d**-**i** Cutting–healing process of the as-fabricated self-healable EL device. **d**-**ii** Schematic of the cut region, which illustrates the self-healable mechanism and the configuration of each layer. **e** SEM image of the initial PU phosphor layer from bottom to top view angle. **f** SEM image of healed PU phosphor layer; the healed wound was represented by the red rectangle

Targeting the realization of a self-healable EL device, the physicochemical properties of each functionalized component layer should be considered. The synthesis process of the PAA layer and PU composite layer is shown in Fig. 1a and Fig. 1b, respectively. The polymer-based hydrogels have been well developed for broad applications via rational design at the molecular level to obtain improved physicochemical characteristics, such as visible-light transparency, ionic conductivity, and self-healability. Compared with film or bulk-shaped self-healing electrodes, which are based on conductive composites for EL devices, where high transmittance, conductivity, and healability are required for conductive frameworks and supporting frameworks, hydrogel was selected for simultaneous realization of these properties^21^. Considering the requirements for the electrode layer in EL devices, we chose modified PAA hydrogel as a self-healable electrode based on the following reasoning: PAA hydrogel that contains 4 M NaCl can achieve 96.5% average transmittance in the range of visible light (wavelength that ranges from 380 to 800 nm) and a conductivity of 2.1 S m^−1^ (Figure S1, S2). The uniform distribution of NaCl crystals in freeze-dried PAA/hydrogel verified the highly ionic conductivity of the PAA/NaCl electrode (Figure S3). In addition, the carboxyl groups on PAA backbones endow the hydrogel with self-healing capability via hydrogen bonding, which aims to restore these physicochemical properties of PAA hydrogel after deadly damage^18,29^. Regarding the phosphor composite layer, it must be electrically insulating and self-healable. We chose PU modified with carboxyl groups as a guest-hosting binder for nanoparticles, which enables ZnS phosphor particles to physically disperse in the PU polymer matrix, to form a self-healing light-emitting composite layer. The synthetic routes of PU are shown in Figure S4. Boron nitride (BN) nanosheets were chosen as a dielectric enhancement additive that was aimed at higher dielectric permittivity of the phosphor layer, and consequently, enhanced luminescence^30^. Healability of the composite layer can be achieved by hydrogen bonding between modified carboxyl groups on PU backbones^27,31^.

The designed EL device is a three-layered configuration that consists of an EL dielectric phosphor composite layer that is sandwiched by two symmetrical layers of ionically conductive hydrogel electrodes, as shown in Fig. 1 c–i. Metal-based electronic conductors were attached to the hydrogel layers, which produced electrical double layers (EDLs). The EDL area (A_EDL_) is only a small portion of the entire area of hydrogel (A_H_), which guarantees the stability of hydrogel by avoiding interfacial electrochemical reactions^7,9,32^. An equivalent circuit of the EL device is shown in Fig. 1 c-ii, where the EDL and dielectric layer behave as capacitors in series. To evaluate the performance of the circuit, these capacitors are assumed to be linear. Thus, Q = C_1_V_1_ = C_2_V_2_ = C_3_V_3_, where Q is the amount of charge and C and V represent the corresponding capacitance and voltage, respectively, of each layer. The capacitance of the PU-based dielectric layer is considerably smaller (C_2_/C_1_ = 10^−5^–10^–4^) than that of the EDL in a lower frequency range, as shown in Figure S5, which indicates that the applied voltage is concentrated on the phosphor layer. In addition, the net capacitance of the circuit is dominated by the dielectric layer, whose capacitance can determine the final distribution of the applied voltage on the phosphor layer, which determines the luminescent intensity of the EL device. The emitted light is a result of the recombination of excitons within the intrinsic heterojunction of ZnS phosphor powders in an alternating current electric field, whereas the light wavelength is tuned by dopants in the ZnS lattice and the voltage-induced luminance by the ionic conductor does not require electrons injected into ZnS phosphor particles^2,33^. In addition, the electronic structure of the dielectric layer as the host matrix would not affect the excitation mechanism of ZnS particles, whereas the higher permittivity of the dielectric layer would lead to the concentration of the applied voltage on the phosphor particles. Detailed discussions about the mechanism of the EL device are presented in the theory part of the supporting information.

The self-healing process of the EL device is illustrated in Fig. 1d. The device was cut, and the resultant fresh wounds were gently pressed into contact. The detailed structure at the cut region is shown in zoomed-in Fig. 1 d-ii. Due to the sedimentation of ZnS particles during solidification, the phosphor layer stratified into two parts. In the lower part, ZnS particles are stacked and BN nanosheets are bound by PU located in the interstitial spaces among the ZnS particles. In the upper part, BN nanosheets uniformly dispersed in the PU matrix, as illustrated in the magnified image in Fig. 1 d-ii. The SEM images distinctly revealed this sedimentation phenomenon of the PU layer in Figure S6, and the distributions of each component in the phosphor layer were verified by energy- dispersive X-ray analysis (EDX) in Figure S7. The sedimentation of phosphors can be avoided if organic polymers were introduced to the emissive layer, whereas some challenges are encountered in selecting organic phosphors with the capability to excite sufficient carriers of polymer phosphors embedded in the dielectric host matrix for alternating current EL devices. In addition, the morphology and size of the ZnS and BN nanosheets are shown in Figure S8. Due to the relatively large size of ZnS particles, the mechanical strength of the ZnS/BN/PU composite layer after healing is slightly affected as the majority of the space was occupied by the stacked ZnS particles instead of the self-healable PU matrix. For the upper part of the phosphor composite layer, the mechanical strength can be well restored due to the large proportion of the healable PU matrix, which accounts for the mechanical performance recovery for the entire phosphor layer. The morphology of the initial and healed region of the phosphor composite layer was observed, as shown in Figs. 1e–1f. PU polymer from two different cut units healed as a unit, whereas the remaining a fosse line was created during the cutting procedure.

### Self-healing performance of each EL component layer and the device

**Fig. 2:**
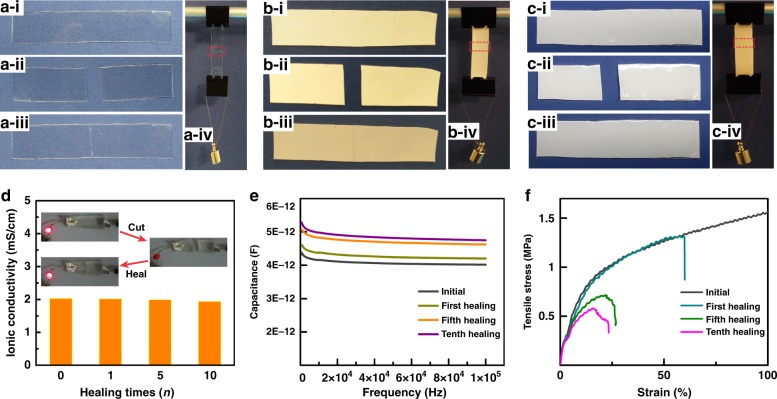
Self-healing properties of each functional layer and the device. **a–c** Images of the self-healing processes of the EL component layers and the device (PAA layer—**a**, PU composite layer—**b**, and EL device—**c**). In these processes, **i** shows the initial states, **ii** shows the cut states, **iii** shows the healed states, **iv** shows the healed states by hanging a weight, and the red rectangle denotes the healed wounds. The weight is 10 g of mass. **d** Ionic conductivity of PAA conductor after multiple cutting–healing times. **e** Dielectric capacitance of PU phosphor layer after multiple cutting–healing times. **f** Mechanical strength of the EL device after multiple cutting–healing times

Optical images of different stages of the initial-cut-healed procedure (refer to details in Materials and Methods) for the PAA-based ionic conductor layer, PU-based phosphor layer, and the assembled EL device are exhibited in Fig. 2a–c, where the restoration of mechanical strength after healing for each layer is evaluated by hanging a weight. In addition, the multiple cutting–healing procedure, which refers to ten repeated cutting–healing cycles for each layer, and the device are shown in Figure S9-S11. Considering the distinct roles of each functional layer in the EL device, the physicochemical properties of the individual layer after healing were investigated prior to evaluating the performance restoration of the assembled EL device. For the PAA-based ionic conductor, the ionic conductivities were measured by the electrochemical impedance spectroscopy (EIS) (Figure S12). They were almost fully restored compared with the initial value, even after ten cutting–healing cycles (Fig. 2d). We also observed the reviving process of a light-emitting diode (LED) circuit after healing of PAA hydrogel (inset of Fig. 2d). The PU-based dielectric phosphor layer, which sustains the majority of the applied voltage, is required to restore its dielectric permittivity and capacitance. As shown in Fig. 2e, the capacitance of the dielectric layer remained almost stable after different healing cycles with only 16.8% increase in capacitance at 1000 Hz even after ten cutting–healing cycles, which indicates that the distributed voltage on this layer and the corresponding light-emitting intensity after healing would be almost constant. The mechanical properties of the healed EL device are well studied with regard to healing times, which restored 537 kPa of the tensile strength and 2.4 MPa of the Young’s modulus at the device breaking point even after ten cutting–healing cycles (Fig. 2f and Figure S13). The restoration of mechanical strength endows the EL devices with undiminished mechanical flexibility after healing. Information about the mechanical properties restoration after different cutting–healing cycles for individual layers, which are also provided in Figure S14, indicates that the PAA- and PU-based layers can sustain their mechanical strength after healing the cut wound. In addition, recovered mechanical strengths with different healing times have also been evaluated. The findings revealed that the tensile stress increased with an increase in the healing time both for the electrode layer and the EL layer and tended to stabilize after 30 min (Figure S15). The ionic conductivities of hydrogels with different NaCl concentrations were also measured, and the corresponding tensile stress at the fracture points exhibited no distinct variation, which indicates that the self-healing properties were determined by the polymer framework of a hydrogel (Figure S16).

### Light-emitting performance of the self-healable EL device

**Fig. 3:**
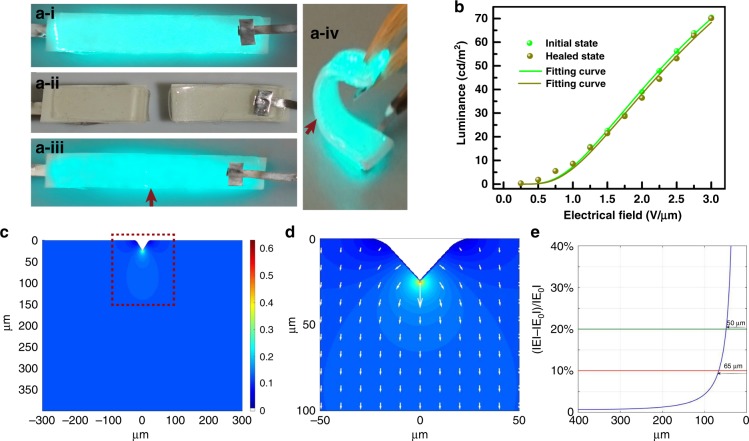
Light-emitting performance of the self-healed EL device after cutting–healing process. **a** Demonstration of cutting–healing process of the healable EL device. **a-i–a-iii** exhibit the initial working state, cut state, and revived working state, respectively, after healing. **a-iv** shows the bend working state of the healed EL device. **b** Luminance–voltage characteristics of the EL device for the initial and healed states. **c** Distribution of the electrical field across the phosphor layer, where the color bar represents the intensity of the electrical field. **d** Magnified image of a marked region in **c**, where the lengths and directions of the arrows represent the values and directions, respectively, of the electrical field adjacent to the healing region. **e** The value variation of the electrical fields as a function of the distance to the healing point, where ΙEΙ represents the values of the redistributed electrical fields and ΙE_0_Ι represents the original value of the distributed electrical fields before the cutting–healing process

Guided by the rational design for both the materials and the device, the resultant EL device was demonstrated in the initial working state, cutting state, and healed state (working state after healing), as shown in Fig. 3a. After healing, the three layers of the EL device were recovered, and the EL device was revived, with a restored mechanical flexibility which can sustain severe bending at the angle of 120° (Fig. 3a–iv). The healed region is denoted by red arrows in Fig. 3a iii–iv. Note that the luminance of the self-healed EL device was uniform (Figure S17) and showed slight intensity decay (6.7%) after healing even when subjected to deadly cuts, where the driven voltage at the marked spot remained identical (2 V/µm, 500 Hz). This successful reviving of the light-emitting performance for the EL device can be attributed to the restoration of each functional layer in the mechanical and physicochemical properties. The luminescent performance after the first healing process was further investigated when the EL device was subjected to tensile stress, in which the healed device was able to maintain luminance to the failure point of the device at 1.21 MPa with a strain at 41.6% (Figure S18), which can support the mechanical strength and flexibility of the EL device (Fig. 3a). The luminance properties were investigated for the initial working state and healed state as a function of applied voltage. The EL devices follow the equation *L* = *L*_0_ exp(–β/*V*^1/2^), where *L* is the luminance, *V* is the voltage, and *L*_0_ and β the constants determined by the materials and the device, respectively. The experimental data fit well with the equation for both the initial working state and the healed state, which indicates that the physical breakage did not substantially affect the constants (*L*_0_ and β), as shown in Fig. 3b. The luminance performance of the EL devices with different electrode conductivities of electrodes showed slight variation with identical driving voltage (Figure S16). The EL device can achieve decent luminance of 70.5 cd/m^2^ at 3 V/µm and 500 Hz. The small intensity variation may be caused by the redistribution of the electric field applied on the phosphor layer due to the slight configuration transmutation of the healed region during the cutting–healing process. To examine the mechanism for light intensity variation, we further examined the redistribution of the electrical field in the healed region of the phosphor layer before and after the self-healing process with the finite difference method, as elaborated in the Calculation section in supporting information, as to the applied voltage is dominantly distributed across the phosphor layer, which determined the intensity of the emitting light. The model was established using the realistic shape deformation after the cutting–healing process of the phosphor layer, where a sunken spot (depth of ~20 μm) was created, as shown in Figure S19. The redistribution of the electrical field across the phosphor layer is shown in Fig. 3c. The electrical field varied adjacent to the healing point but the variation dissipated within the space span (Fig. 3d). Specifically, the electrical field is five times higher at the healing point and quickly decays to only 20% (10%) higher within 48 μm (65 μm) from the uncut top plate (Fig. 3e), where the electrical field at the top plate is regarded as identical to the original value prior to the cutting–healing process. Consequently, this result verified the variation of luminance adjacent to the healing region and explained the possible origin for degradation of the luminance after healing. These results have also provided a solution for improving the performance of healable EL devices by avoiding shape deformation and misalignment during the cutting and healing process. The entire process of an EL device in the initial working state, cutting state, and healed state can be observed in movie S1.

### Robust vitality of the self-healable EL device

**Fig. 4:**
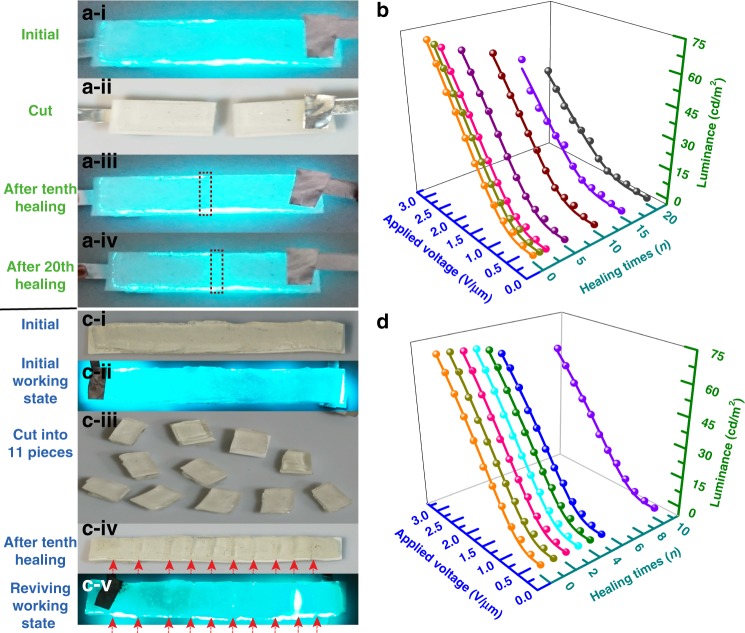
Self-healable EL device subjected to different cutting modes. **a**, **c** demonstrate photographs of the EL device subjected to multiple cutting–healing times in FCM. **b**, **d** exhibit the relationships between luminance–voltage-healing times of the self-healable EL device throughout FCM and UFCM at 2 V/µm and 500 Hz, respectively

Encouraged by the presented results, we further investigated the multiple healing ability of the EL device by adopting two cut modes, i.e., fixed cut mode (FCM) and unfixed cut mode (UFCM), which refer to cutting at the same spot of the device and cutting at different spots of the device, respectively. The luminance of a marked spot on an EL device was measured during the two cutting modes to characterize the light-emitting intensity and evaluate the multiple self-healing efficiency. The 20 cutting–healing cycles of the EL device are demonstrated in Fig. 4a, where the initial working state, cut state, and healed state after 10 and 20 cutting–healing cycles are shown in Fig. 4 a-i–iv, where the red rectangles denote the healing spots. The corresponding luminance curve is examined in Fig. 4b as functions of applied voltage and cutting–healing cycles. The luminance intensity degrades with an increase in the cutting–healing cycles, and the healing efficiency of luminance after 20 cutting–healing cycles in FCM remains 57.7%. Images of the EL device that undergoes UFCM are exhibited in Fig. 4d. The EL device remained in one piece and properly worked in the initial working state (Fig. 4d-i, ii) until it was subjected to 10 cuts, which converted it into 11 individual pieces. All pieces were healed together into one piece, as shown in Fig. 4d-iii, iv. The healed wounds are dimly observed on the device surface due to the misalignment between the cross-sections during healing. The healed state after ten cutting–healing cycles in UFCM was demonstrated in Fig. 4d–v. The red arrows in Fig. 4d-iv–v pointed out the healing regions. The luminance after different cutting–healing cycles as a function of voltage is analyzed in Fig. 4e and the healing efficiency for ten cutting–healing cycles in UFCM is 83.2%. The corresponding tendencies of the healing efficiency for the FCM and UFCM are shown in Figure S20. The reason for the degradation in luminance after these two catastrophic tests is discussed here. The polymer-based materials suffer irreversible shape deformation to some degree during cutting, as illustrated in Figure S21. Thus, this phenomenon will produce a slight shape deformation in the cut region and the subsequent healed region. In our case, PAA and PU polymer underwent different cut modes, and shape deformation accumulated at each cut. Manual operation will potentially cause misalignment in the cut regions during healing. Therefore, the unavoidable shape deformation and manual operation will cause the redistribution of the applied electric field and affect the luminance after multiple cutting–healing processes. Considering the minor decay of luminance after either cut mode, the device can maintain its performance after tens or even hundreds of cutting–healing cycles in mixed cut modes. Some approaches may improve the healing efficiency of the EL device, such as developing a self-healable phosphor with surface ligands and more exquisite techniques for the self-healing process.

### LEGO assembly of the self-healable EL devices

**Fig. 5:**
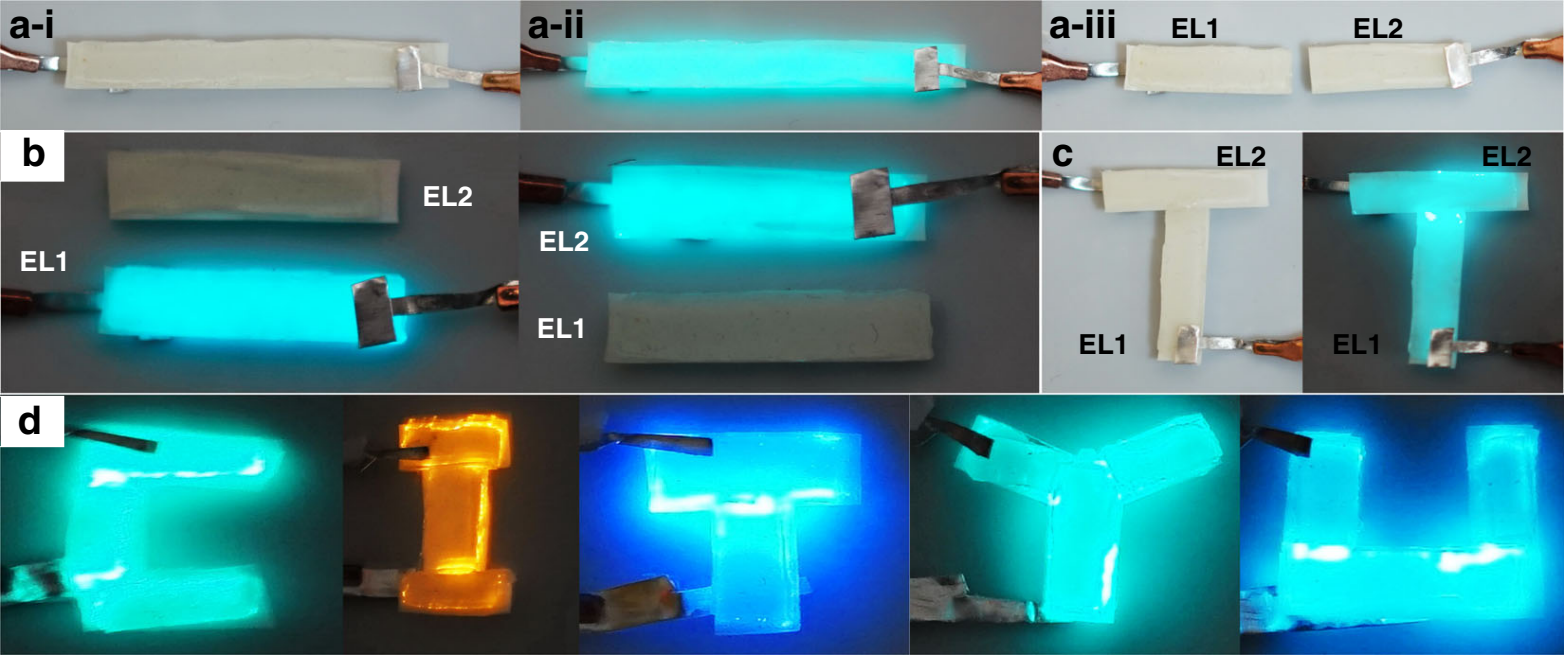
LEGO assembly process of self-healable EL devices. **a** A complete EL device was cut into two individual EL units. **a-i** indicates the initial unworking state, **a-ii** the working state, and **a-iii** the cut state. **b** The as-obtained two EL units in working state. **c** The as-obtained two EL devices were assembled into a “T” light-emitting letter. **d** The assembled light-emitting characters as “CITYU” with different EL units

From the outstanding healability of the as-fabricated EL devices, a few EL units can be assembled into one integrated EL system, which realizes a “LEGO” assembly of light-emitting devices. Every piece of an EL unit after cutting remains a complete EL unit, which comes in a smaller size, as the sandwiched configuration of the device remains, and each layer preserves their properties. Specifically, the initial EL device was cut into two individual EL units, namely, EL1 and EL2 (Fig. 5a), and both EL units properly work without any visible luminance degradation (Fig. 5b). The “LEGO” assembly process was implemented by assembling two EL units into a ‘T’ light-emitting characteristic of the healability of all functional layers in our EL device (Fig. 5c). We further demonstrated the conceptual “LEGO” assembly by presenting various EL-assembled units into a “CITYU” logo with colorful emitting lights (Fig. 5d, S22) by applying ZnS phosphor particles with different doped elements. This study is the first study that has reported an arbitrary assembly of light-emitting devices.

## Discussion

This study demonstrated the first self-healable EL devices, in which all functional layers are healable. The physicochemical and mechanical properties can be well restored for both individual layers and full devices even after experiencing multiple catastrophic damage events (i.e., dozens of cutting–healing cycles). The performance restoration is attributed to the rebuilding of hydrogen bonds during healing in each component layer. As a result, the issue of fractures induced by mechanical deformation is addressed and the luminance performance of the EL device can be subsequently restored with a high healing efficiency (83.2% for 10 healing cycles at unfixed spots and 57.7% for 20 healing cycles at a fixed spot). Note that the novel design of our self-healable EL device can be readily applied to develop other ionically conductive healable hydrogels as transparent electrodes and healable dielectric polymers as phosphor hosting layers, which can considerably enhance the reliability and vastly broaden the application of EL devices in wearable and deformable electronics. In addition, the impressive healability of the developed EL devices was further evolved to demonstrate the assembly of a few EL units into one light-emitting system, namely, the “LEGO” assembly, for the first time. This assembly protocol is expected to have an economic role in repairing and replacing light-emitting modules in complex integrated electronic systems.

## Materials and methods

### Materials formulation

Unless they were specified, the materials were used as received without further purification. Polyacrylic (PAA)/NaCl hydrogel roles as ionic conductor and polyurethane (PU)/zinc sulfide (ZnS)/boron nitride (BN) nanosheets composite polymer matrix roles as the phosphor layer.

The hydrogel was synthesized using acrylic acid (AA; Acrocs Organics, Code: 164250010) as monomers, N,N-methylenebisacrylamide (MBAA; Aladdin, Code: M104026) as cross-linkers, and ammonium persulfate (APS; Acrocs Organics, Code: 401165000) as thermal initiator. AA and NaCl were dissolved in deionized water in an ice bath fixed at 5.1 M and 4 M. APS was added to the solution by 0.0152, the weight of AA, and MBAA was added to the solution by 0.0005, the weight of AA. The solution was vigorously stirred for 30 min and degassed in a vacuum chamber. The obtained solutions were poured into a 100 × 100 × 0.4 mm^3^ glass mold and covered with a thick glass plate. The mold was deposed in a 70 °C oven for 40 min. This curing mold was wrapped with Al foil paper to ensure an even heat distribution during the thermal curing procedure. The obtained PAA hydrogel was placed in a humid box for 60 h to stabilize the reactions. After the curing step, the PAA was cut into the designed shapes to act as an ionic conductor for the EL device.

ZnS phosphor powders were purchased from Shanghai KPT Co. Hexagonal BN (h-BN) powder was purchased from Zibo Jonye Ceramic Technologies Co., Ltd., in Shandong province, China. BN nanosheets were fabricated by liquid-phase exfoliation with the assistance of an ultrasonic bath KX-1740Q1 at 120 W. In a typical run, 1.0 g of pristine h-BN micro-powder was added to 300 ml of water and sonicated for 5 h. After the sonication, the whitish suspension was kept constant for 15 min, and then the supernatant was collected via centrifuging at 1500 rpm for 10 min while the sediment was disposed. The hydroxylated BN nanosheets were obtained by refluxing the BN nanosheets with over-amount hydrogen peroxide at 100 °C for 12 h. The final product was collected by filtering and then dried at 100 °C prior to future use.

The carboxylated polyurethane (PU) was synthesized using polyethylene glycol (PEG; Aladdin, Code: P103731), 3-isocyanatomethyl-3,5,5-trimethylcyclohexyl isocyanate (IPDI; Aladdin, Code: I109582), dibutyltin dilaurate (DBTDL, Aladdin, Code: D100274), and dimethylol propionic acid (DMPA, Aladdin, Code: B104539). A total of 0.62 mmol PEG, 12 mmol IPDI, and 0.015 mmol DBTDL were added to a dried vessel with 50 mL of dimethylformamide (DMF) at 90 °C oil-bath reaction for 3 h with mild mechanical stirring in a N_2_ atmosphere. A total of 8 mmol DMPA dissolved in 25 mL of DMF was slowly added to the vessel as chain extender and reacted for another 2 h in the same condition. After the polymerization of PU, the as-obtained solution was stored for dispersion of ZnS phosphor powders and hydroxylated BN nanosheets. To fabricate the PU-based phosphor layer, in a typical run, 1 g of ZnS phosphor powders and, 0.3 g of hydroxylated BN nanosheets were added to a 30-mL urethane compound with mild stirring for 6 h to obtain uniform dispersion of ZnS powders and BN nanosheets. The mixture solution was poured into a 30 × 30 × 0.4-mm^π^ glass mold and covered with a thick glass plate. The mold was placed flat in a 70 °C vacuum oven for 12 h, whereas the residual solvent was evaporated, and the PU matrix was achieved. To obtain various light-emitting colors, different phosphor particles were utilized, where ZnS was doped with Cu for the green light, ZnS was doped with Cu and Al for the blue light, and ZnS was doped with Mn for the orange light. The obtained PU/ZnS/BN composite layer was cut into the designed shapes to act as a phosphor layer.

### EL device fabrication and characterization

The obtained PU/ZnS/BN composite phosphor layer was sandwiched by two symmetric PAA/NaCl-based transparent ionic conductors. Before the PAA hydrogel was stacked onto the dielectric layer, we use mild flows of nitrogen gases to remove the residual water on the surface to enhance the adhesion between the PAA and the PU layer. The EL device was completed by sticking aluminum conductive tape onto the edge of the PAA electrode to act as the electric conductive electrode. The morphology of each component layer and EDX was characterized by a Hitachi field-emission scanning electron microscope. Each layer (PAA-based ionic electrode and PU-based phosphor layer) was freeze-dried for SEM sample preparation. The optical transmittance spectra of the PAA/NaCl hydrogel was obtained by a UV-visible spectrophotometer (Varian Cary-50 ConC). The ionic conductivity of the PAA layer was conducted by electrochemical impedance spectroscopy (CHI 760D), whereas the ionic conductor was sandwiched between two ITO glass sheets with the dimensions 1.2 cm × 1.1 cm × 0.09 cm (length × width × thickness). The capacitance of the electric double layer and the phosphor layer was measured with a precision LCR meter (Agilent 4284 A), where the measured frequency ranged from 100 Hz to 100 kHz. The restoration of the dielectric capacitance of the PU-based dielectric phosphor layer was evaluated using a fixed-spot cutting–healing process, where the fixed spot was repeatedly cut and healed. The mechanical properties of each layer and the integrated EL device were characterized by the Zwick Z030 tester. Measurements of mechanical strength during the cutting–healing cycles of individual layers and the integrated device were obtained at unfixed spots. The EL device was driven with alternating voltage by a power amplifier (Trek Model 609E-6) and a function generator. The emission light intensity and emission spectra of the device were measured by Spectroradiometer PR650. To evaluate the healing efficiency of the healable EL device, the luminance at marked spots was measured before and after the cutting–healing process at the identical driven voltage. To ensure satisfactory alignment of each layer, the cut EL units were assembled with an optical microscope (Olympus BH2).

### Theory and mechanisms

The electrical and electro-optical characteristics of EL phosphor particles are determined by the host (ZnS lattice), and the emission wavelength is determined by the luminescent center, such as doped elements and defects. Applied by the high electric field (~10 V/µm), the electrons on the luminescent centers were driven into excited states by excited ballistic energy electrons with impact excitation, and the radiative relaxation of the luminescent center subsequently occurred. This process is the basic process for the light emission. At the device level, the ZnS phosphor particles are embedded in a dielectric PU matrix, which can block the electrons to tunnel through the dielectric layer and prevent electrical breakdown at high fields. Thus, the PU-based phosphor layer is only capacitively coupled to the externally applied AC voltage. The capacitive and dielectric properties of the phosphor layer are crucial for the device performance and stability. Below the threshold voltage of the dielectric composite layer to breakdown, our phosphor layer behaves as a capacitor.

For the small contacting area of the Al electrode and the PAA ionic conductor, it acts as an electrical double layer (EDL), where charges are only separated within nanometers; it endows the EDL with large capacitance, where the capacitance is calculated on the order of 10^–1^ F m^–2^. For the dielectric phosphor layer, the thickness is 400 µm and the capacitance is on the order of 10^–7^ F m^–2^. To estimate the behavior of the final voltage distributed at the dielectric layer, we assume that the capacitors as the EDL and dielectric capacitor are linear and in series; thus,

1 $${\mathrm{Q}} = {\mathrm{C}}_{{\mathrm{EDL}}}{\mathrm{V}}_{{\mathrm{EDL}}} = {\mathrm{C}}_{\mathrm{P}}{\mathrm{V}}_{\mathrm{P}}$$

where Q is the amount of capacitively coupled charge, and C, V are the capacitance and the voltage, respectively, for the EDL and the phosphor layer, respectively. This equation indicates that most of the applied AC voltage will be distributed at the phosphor layer to excite the phosphor particles. The luminance is determined by the applied voltage on the phosphor particles. Thus, to stabilize the luminescent performance of EL devices by the cutting–healing process, the voltage distributed on the phosphor layer should not vary after healing. According to equation (1), to stabilize the voltage on the phosphor layer, the corresponding capacitance should stabilize. The mean electric field (E_m_) on the EL cell can be described as E_m _= V/t, where t is the thickness of the phosphor layer. The electric fields on ZnS grains can be described by the following equation:

2 $$E_{{\mathrm{ZnS}}} = E_m\left[ {\frac{{3\varepsilon _P}}{{2\varepsilon _P + \varepsilon _{{\mathrm{ZnS}}} - \lambda \left( {\varepsilon _{{\mathrm{ZnS}}} - \varepsilon _{\mathrm{P}}} \right)}}} \right]$$

where E_ZnS_ is the distributed electric field on ZnS particles; ε_ZnS_ and ε_P_ are the dielectric constant for the ZnS particles and the dielectric phosphor layer, respectively; and λ is the fraction of the total volume occupied by the ZnS particles. According to equation (2), the electric field on individual particles after healing depends on ε_P_, whereas parameters such as ε_ZnS_, E_m_, and λ are assumed to be constant during the cutting–healing process. The luminance of an EL device after healing can be maintained as long as the dielectric capacitance and permittivity remain unchanged.

### Calculation of electric field redistribution at healed spots

To obtain the electrical field $\mathop{E}\limits^{\rightharpoonup} = - \nabla U$ before and after self-healing, the Laplace equation Δ*U* = 0 was numerically solved using the finite difference method, that is, the space is represented by the grid points (i, j), and the potential at each grid point can be updated based on its surrounding potential via the following numerical scheme:

$${U_{n + 1}\left( {i,j} \right) = (U_n\left( {i + 1,j} \right) + U_n\left( {i - 1,j} \right) + U_n\left( {i,j + 1} \right) + U_n(i,j - 1))/4}$$

The boundary conditions are as follows: the potential of the top plate is 100 V, and the bottom top is 0 V. The distance between two plates is 400 μm prior to self-healing. After cutting and self-healing, the top plate is deformed downward by 25μm.

## Electronic supplementary material

Supplementary Information

Movie S1-Self Healable EL device
