# Supplementary material for: Self-healable electroluminescent devices
Source: Light Sci Appl. 2018 Dec 5;7:102. doi: 10.1038/s41377-018-0096-8 (PMC6281662; doi:10.1038/s41377-018-0096-8)
Supplement: Supplementary file 1 — Supplementary Information [file 41377_2018_96_MOESM1_ESM.docx]

Supplementary Information for

**Self-Healable Electroluminescent Devices**

Guojin Liang^1^, Zhuoxin Liu^1^, Funian Mo^1^, Zijie Tang^1^, Hongfei Li^1^, Zifeng Wang^1^, Venkateshwarlu Sarangi^1^, Abhijit Pramannick^1^, Jun Fan^1^*, Chunyi Zhi^1,2^*

^1^Department of Materials Science and Engineering, City University of Hong Kong, 83 Tat Chee Avenue, Kowloon, China. ^2^Shenzhen Research Institute, City University of Hong Kong, High-Tech Zone, Nanshan District, Shenzhen 518057, China.


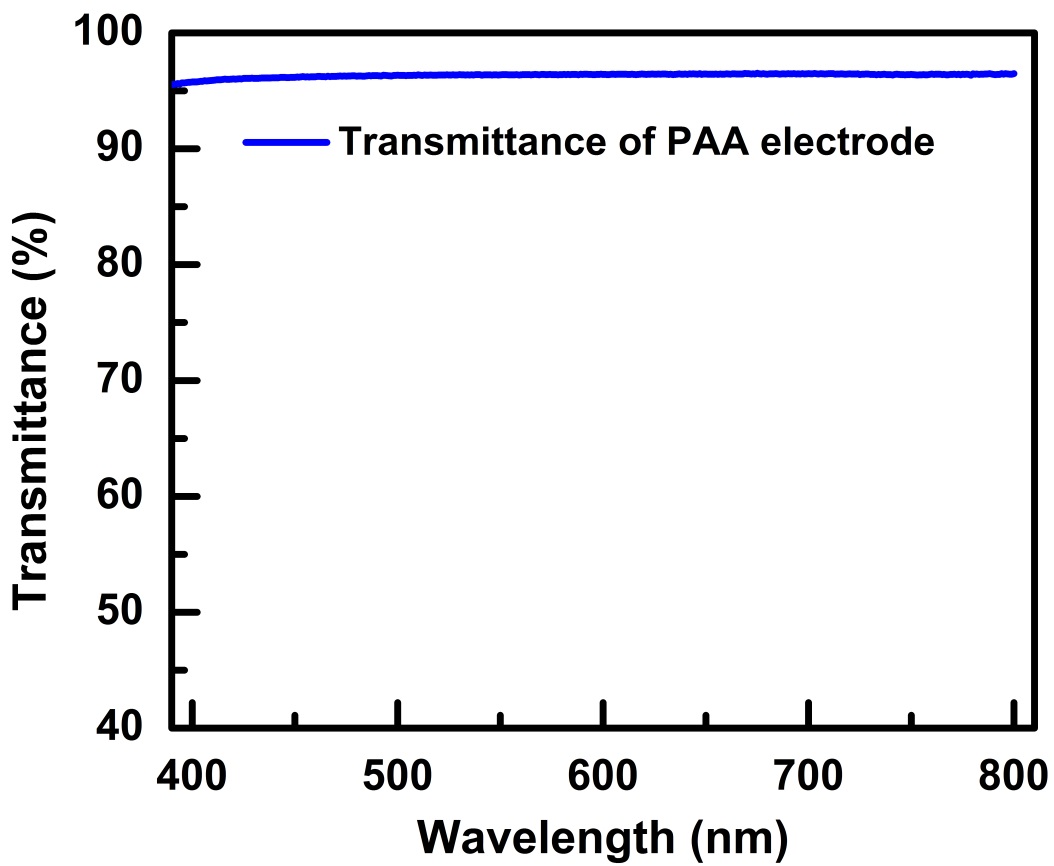


**Figure S1.** A 0.9-mm-thick PAA hydrogel containing 5.1M NaCl shows a 96.4% average transmittance in the visible light range.


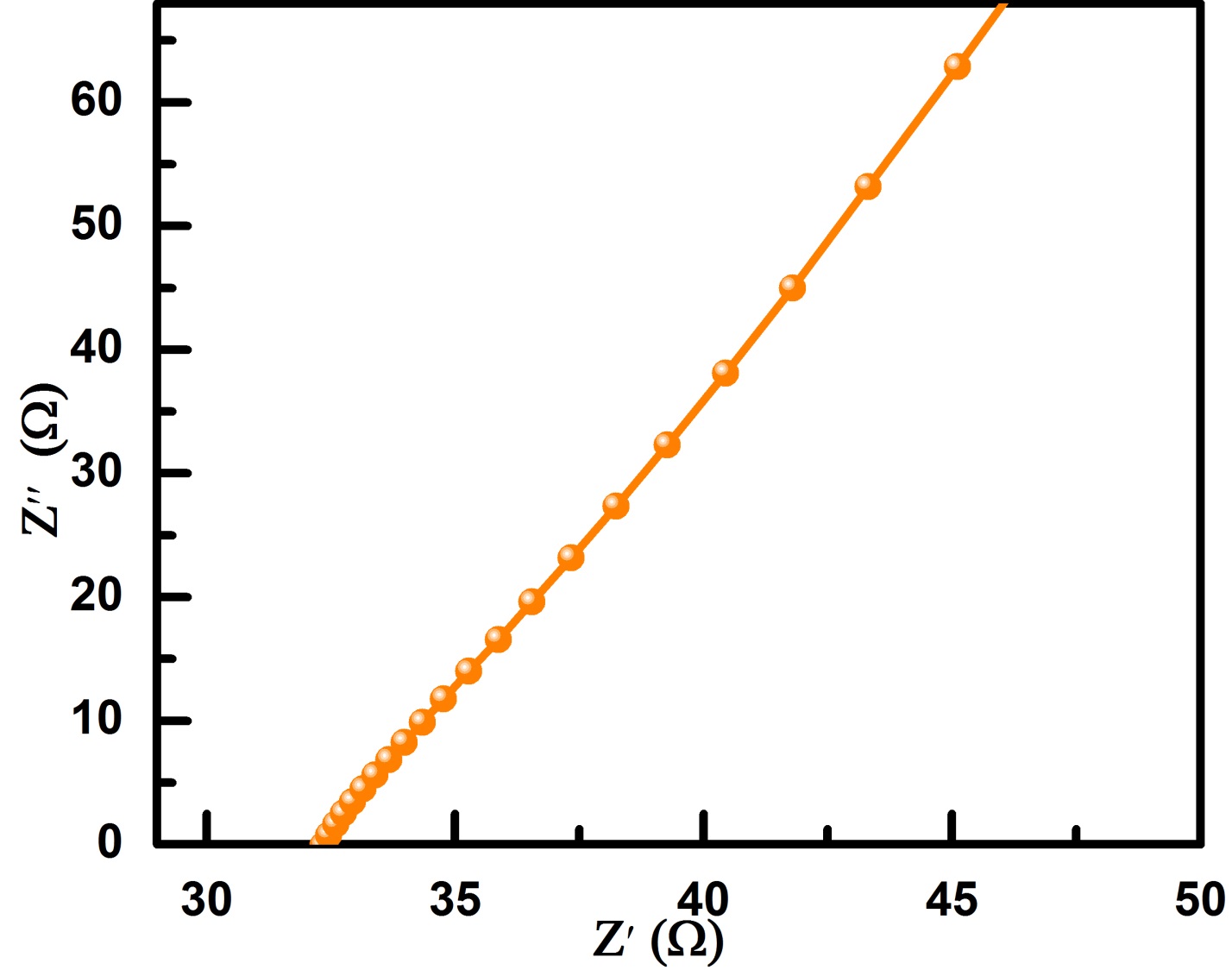


**Figure S2.** The ionic conductivity of PAA hydrogel was measured by electrochemical impedance spectroscopy (EIS), where the PAA sample was sandwiched between two ITO glass with the dimension: 1.2 cm x 1.1 cm x 0.09 cm (length x width x thickness). The high-frequency intercept of the semi-circle in the Nyquist plot can be approximated as the resistance of the ionic conductor. Then, the ionic conductivity is calculated by the following equation:

*σ* = 1**/***ρ* **=** *d***/***RA*

where *σ* is the ionic conductivity, *ρ* is the resistivity, d is the thickness of the ionic conductor, *R* is the resistance, and *A* is the area respectively.


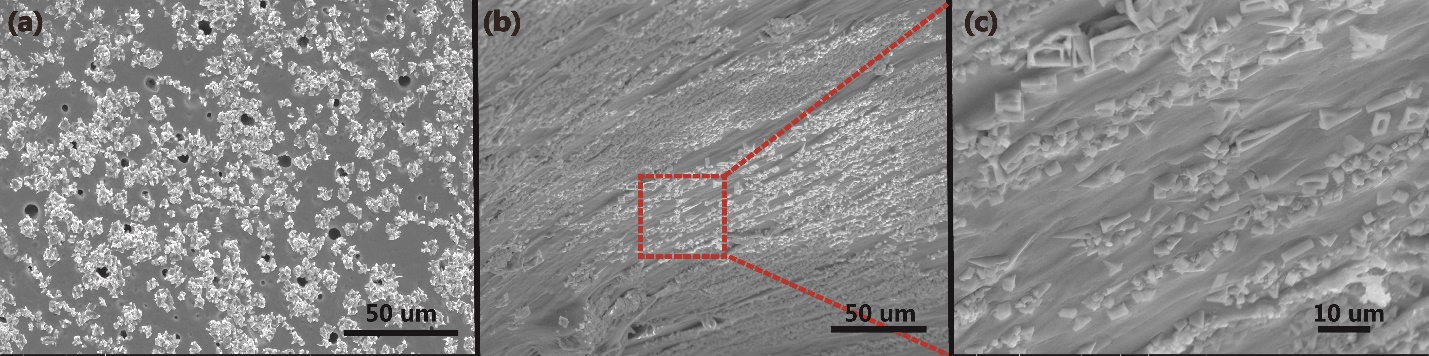


**Figure S3.** SEM images of the freeze-dried PAA/NaCl hydrogel. (a) Uniform distribution of NaCl crystals on the surface of PAA/NaCl hydrogel. (b) Cross-section image of PAA/NaCl hydrogel, where NaCl crystals penetrate uniformly through the ionic hydrogel. (c) Magnified image from (b).


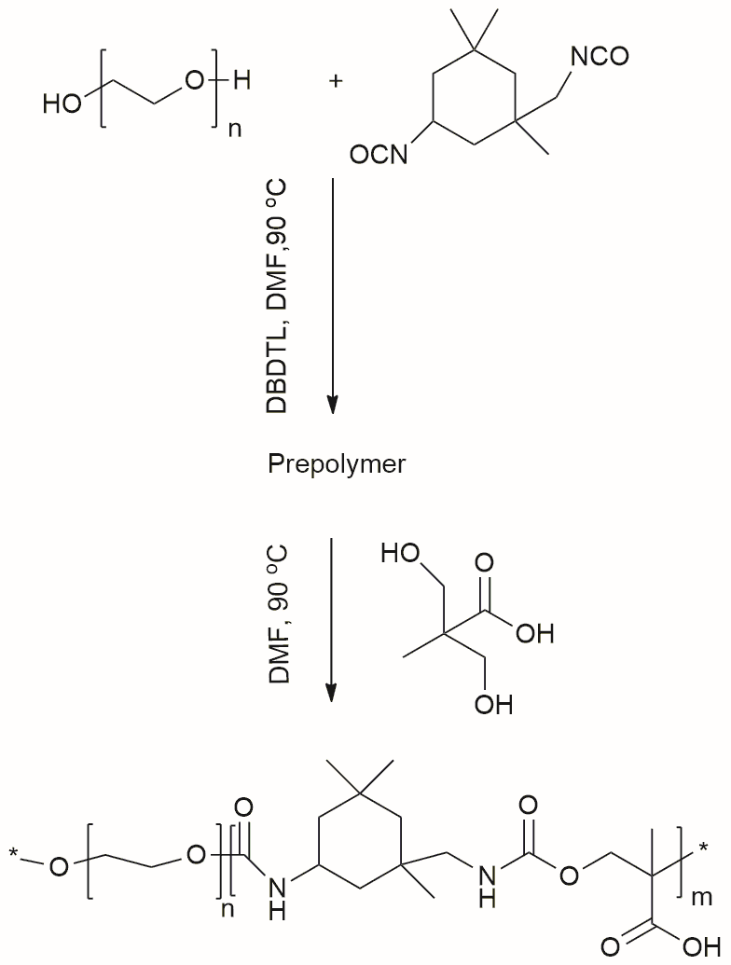


**Figure S4** Synthetic routes of self-healing carboxylated PU.


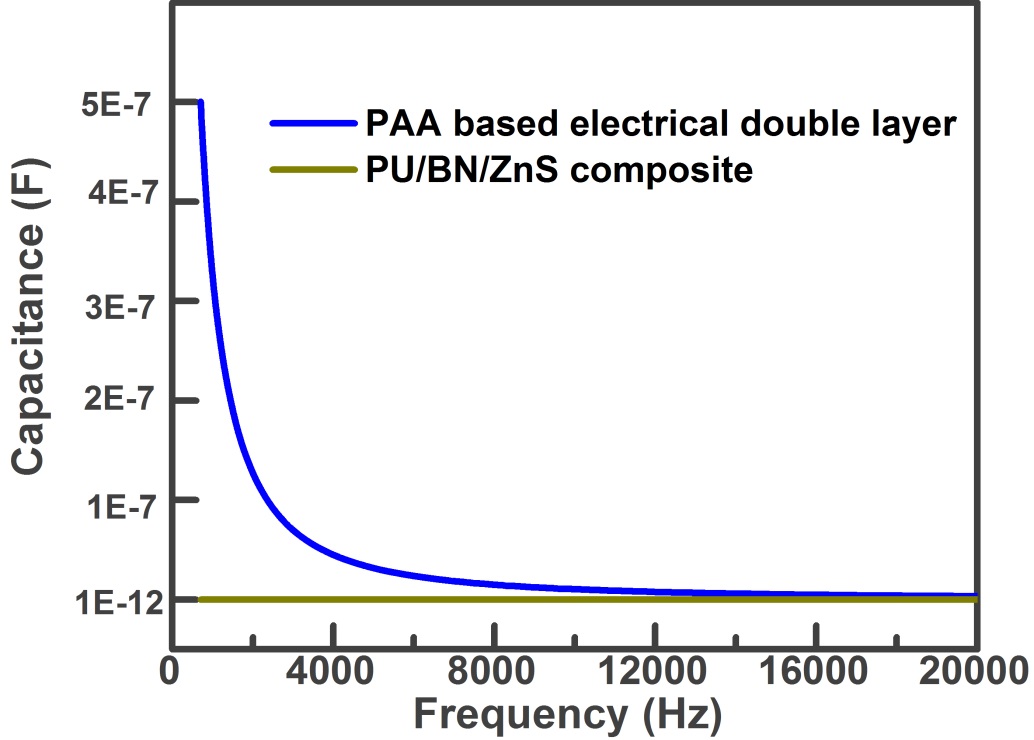


Figure S5. Characterization of the capacitance of EDL and dielectric layer. Because of the same amount of charges stored in these capacitors in series, that is, Q=C_1_V_1_=C_2_V_2_=C_3_V_3_, it verifies most drop of applied voltage is distributed on the dielectric layer.


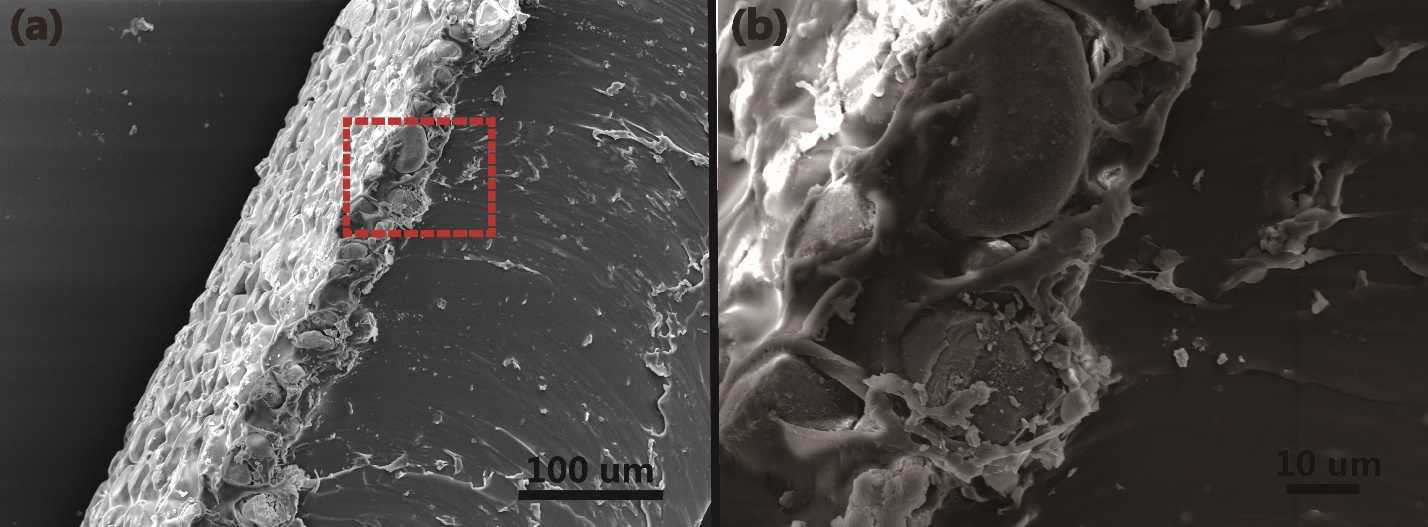


**Figure S6** The cross-section SEM images of the PU-based phosphor layer. (a) Sedimentation of the PU composite layer, where ZnS particles precipitated during the PU solidification process. (b) Magnified image of the red rectangle in (a). In the left part, ZnS and BN were stacked together with PU acting as binder and in the right part, BN dispersed in PU matrix.


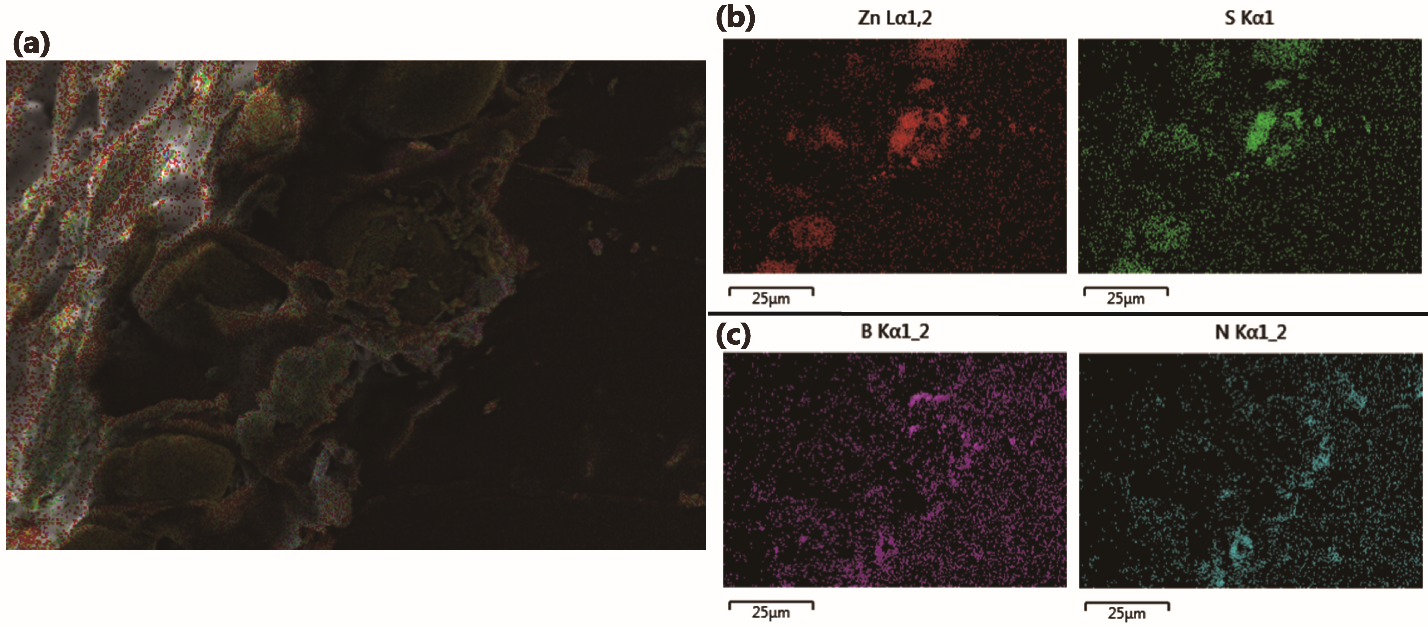


**Figure S7** Dispersive X-ray Analysis of the sedimentation interface of PU composite layer, where locations of ZnS and BN were verified.


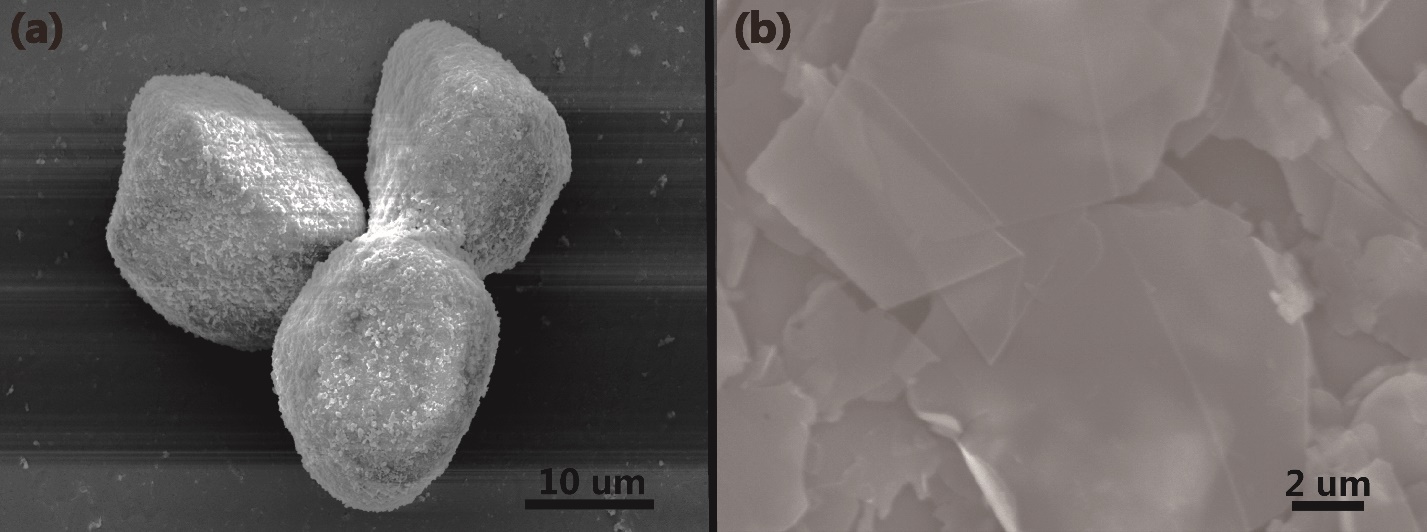


**Figure S8** The morphology and size of ZnS particles and BN nanosheets.


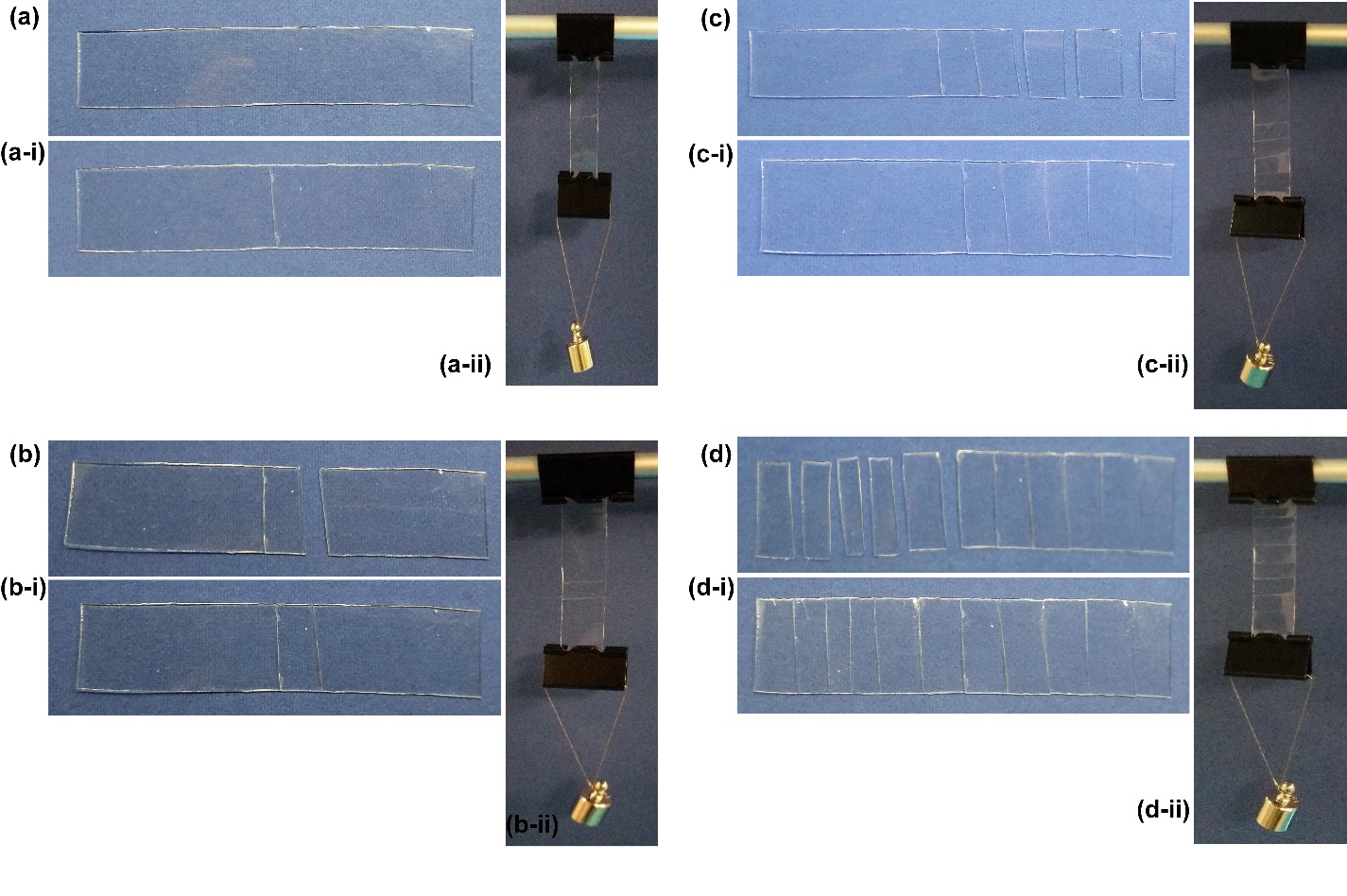


**Figure S9** The process of PAA/NaCl ionic conductor subjected to 10 repeated cutting-healing cycles. (a-d) 1^st^, 2^nd^ ,5^th^, 10^th^ cutting-healing and hanging a weight of healed PAA/NaCl hydrogel respectively.


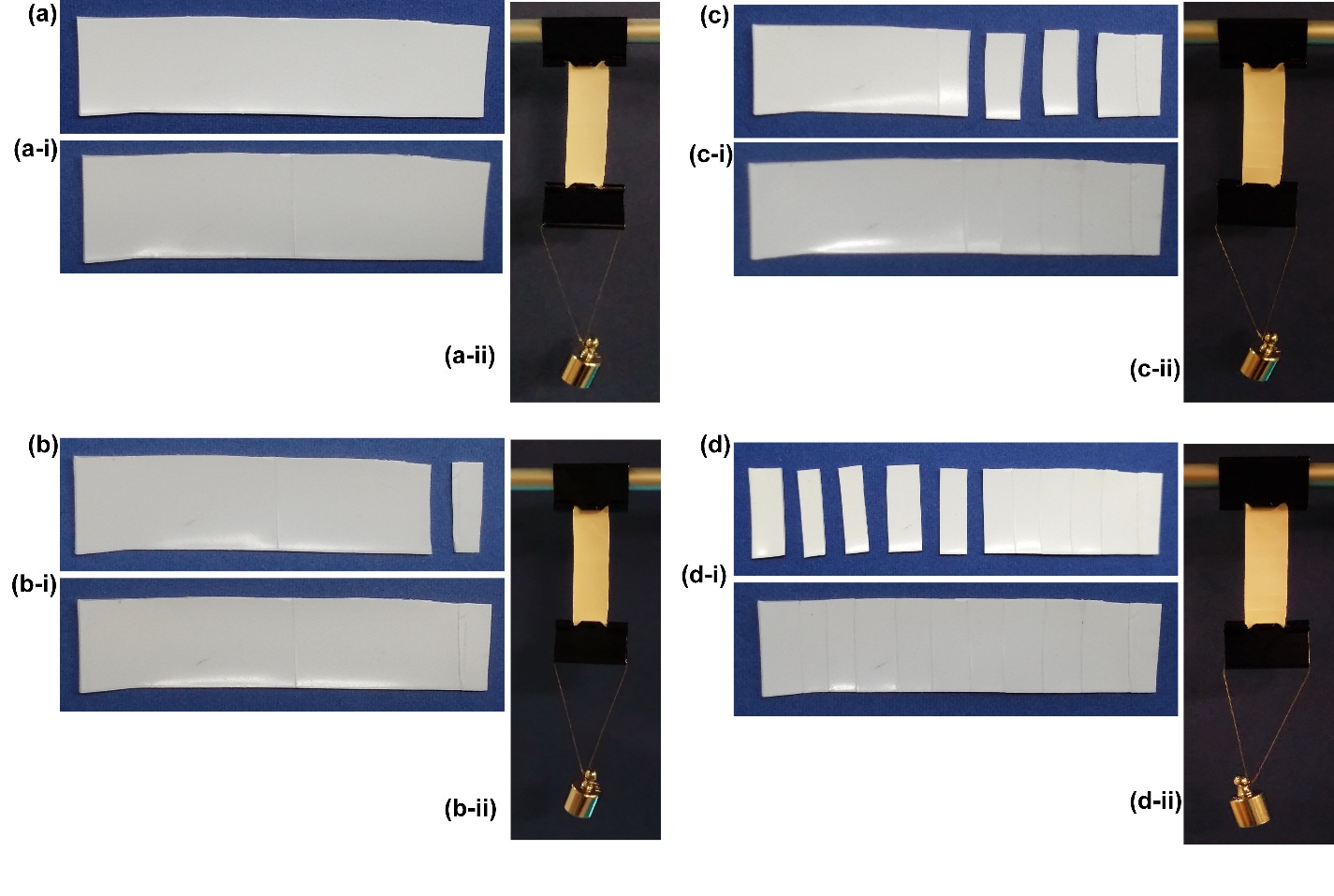


**Figure S10** The process of ZnS/BN/PU composite layer subjected to 10 repeated cutting-healing cycles. (a-d) 1^st^, 2^nd^ ,5^th^, 10^th^ cut, healing and hanging a weight of healed PU-based layer respectively.


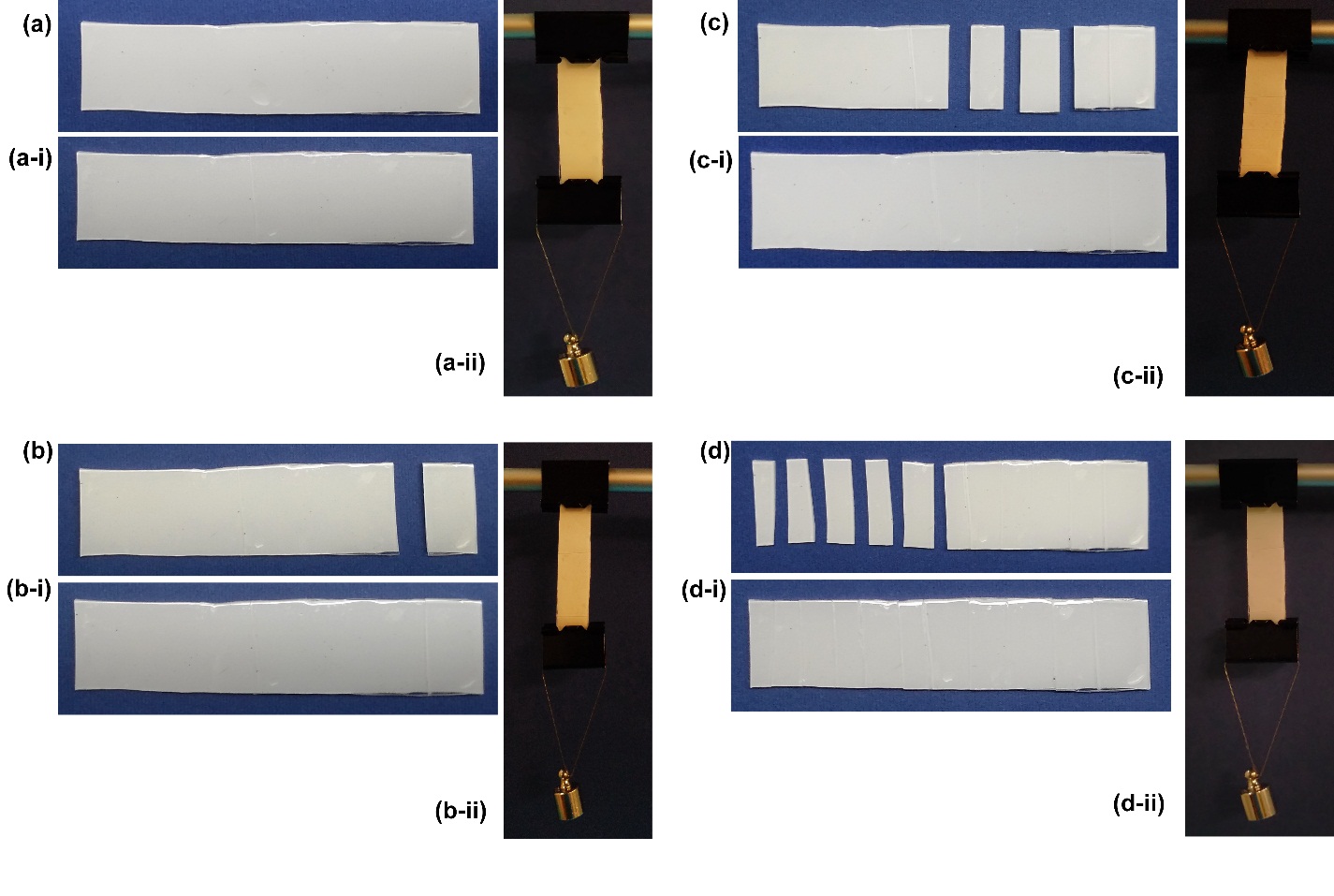


**Figure S11** The process of integrated self-healable EL device subjected to 10 repeated cutting-healing cycles. (a-d) 1^st^, 3^rd^ ,5^th^, 10^th^ cut, healing and hanging a weight of the healed EL device, respectively.


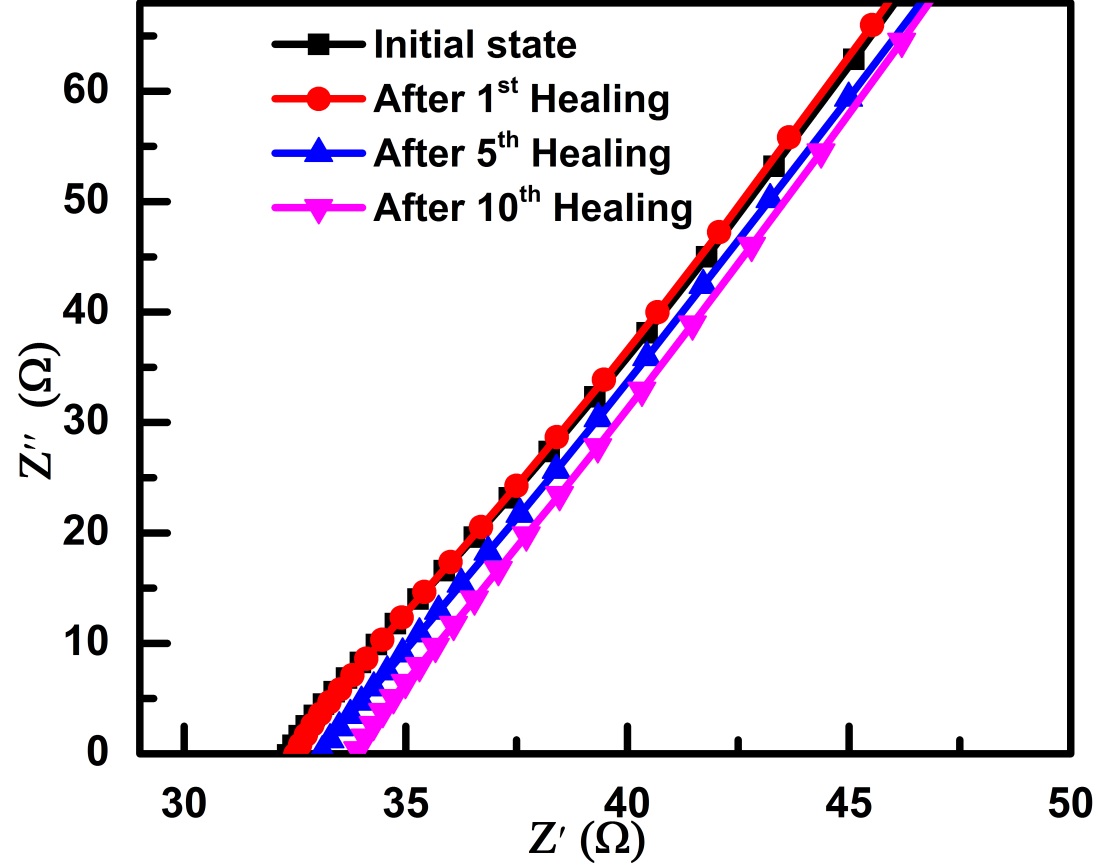


**Figure S12.** The impedance spectra of PAA hydrogel throughout different cutting-healing cycles.


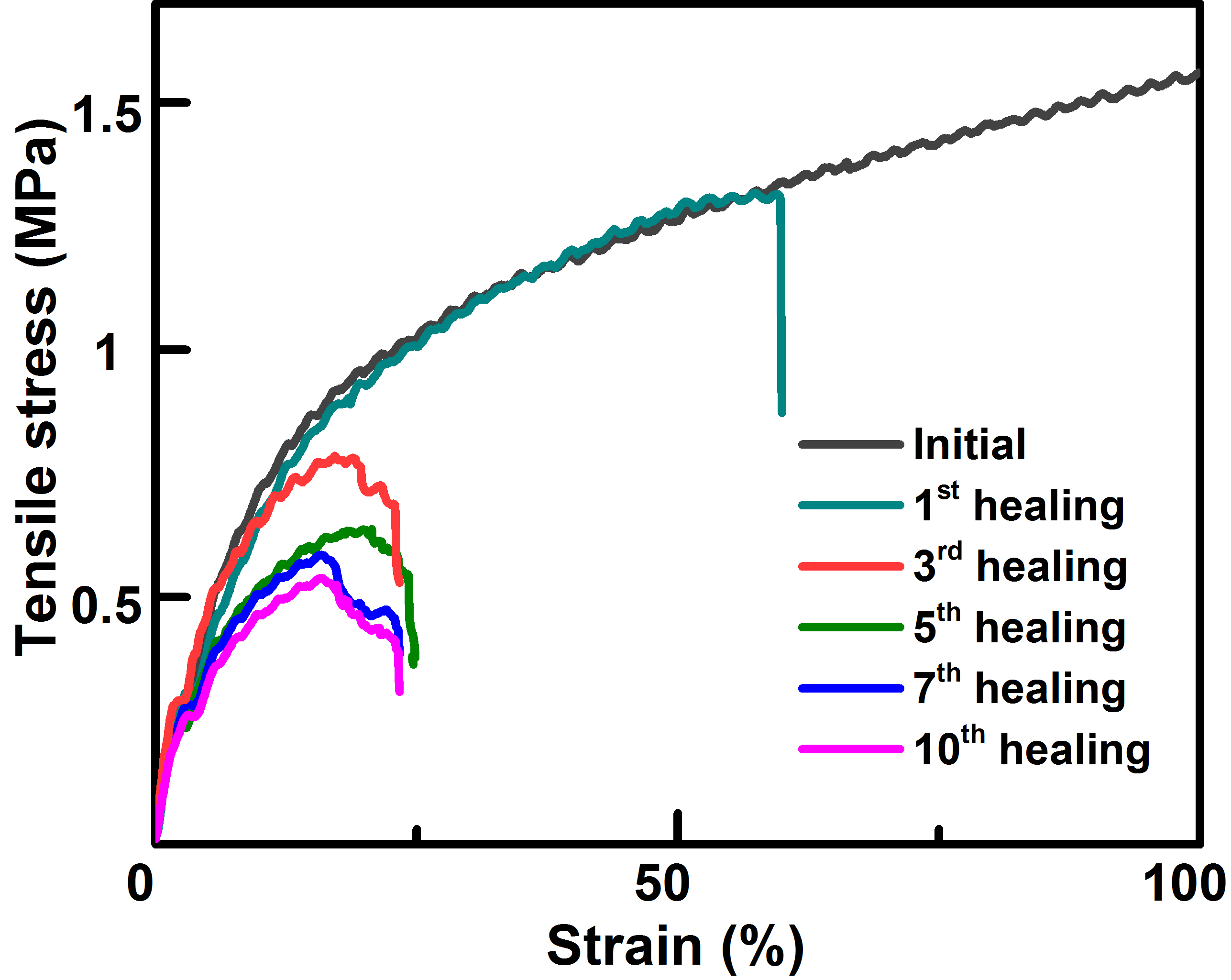


**Figure S13.** The tensile stress of EL device throughout different cutting-healing cycles.


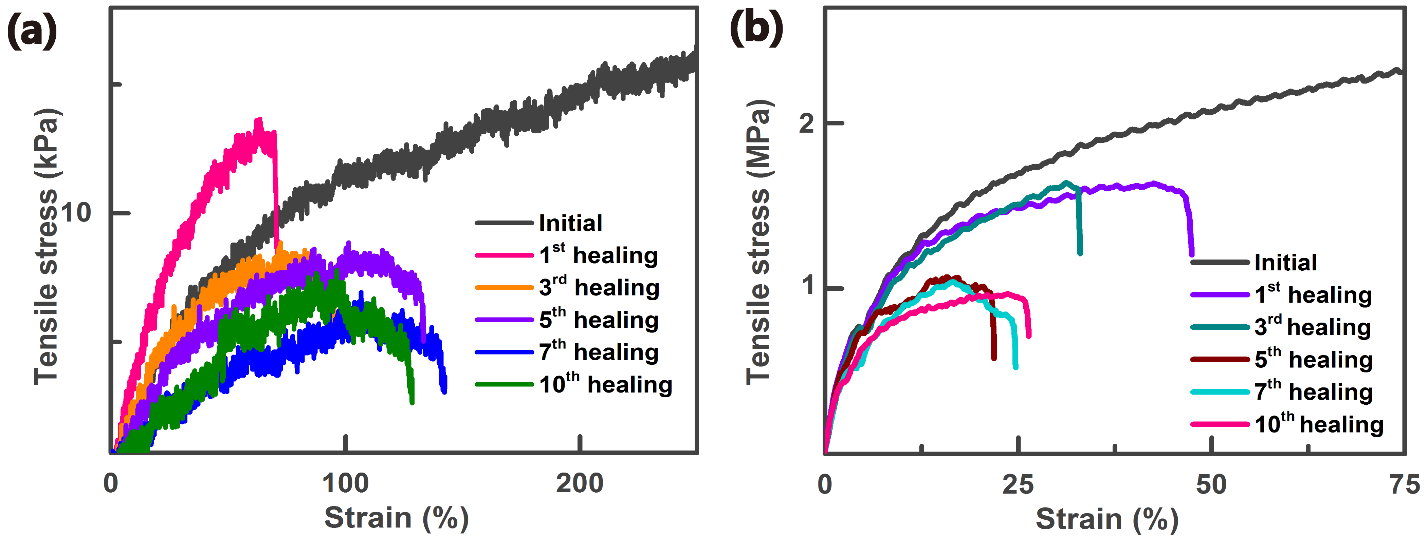


**Figure S14.** Stress-strain curves of the PAA/NaCl hydrogel (a) and PU composite layer (b) after multiple breaking healing cycles. After cutting-healing process, the both healed polymers can sustain tensile stress which guarantee the restoration of mechanical strength for each healed layers, while the elongation fluctuates due to the so-called “barrel effect” and the mechanical strength restoration results depends on the poorest healing result among these multiple healing results during multiple the cutting-healing process. Considering the tensile stress of PU composite layer were approximately 100 times larger than that of PAA/NaCl layer during the mechanical strength tests, it can be understood that the mechanical properties of the healable EL devices are dominated by those of PU composite layer. The restorations of mechanical strength of healable PAA electrodes were beneficial for rebuilding of integrity of ionic conductor, but its contribution to mechanical strength of the devices during tensile tests is limited compared to the role of PU layer.


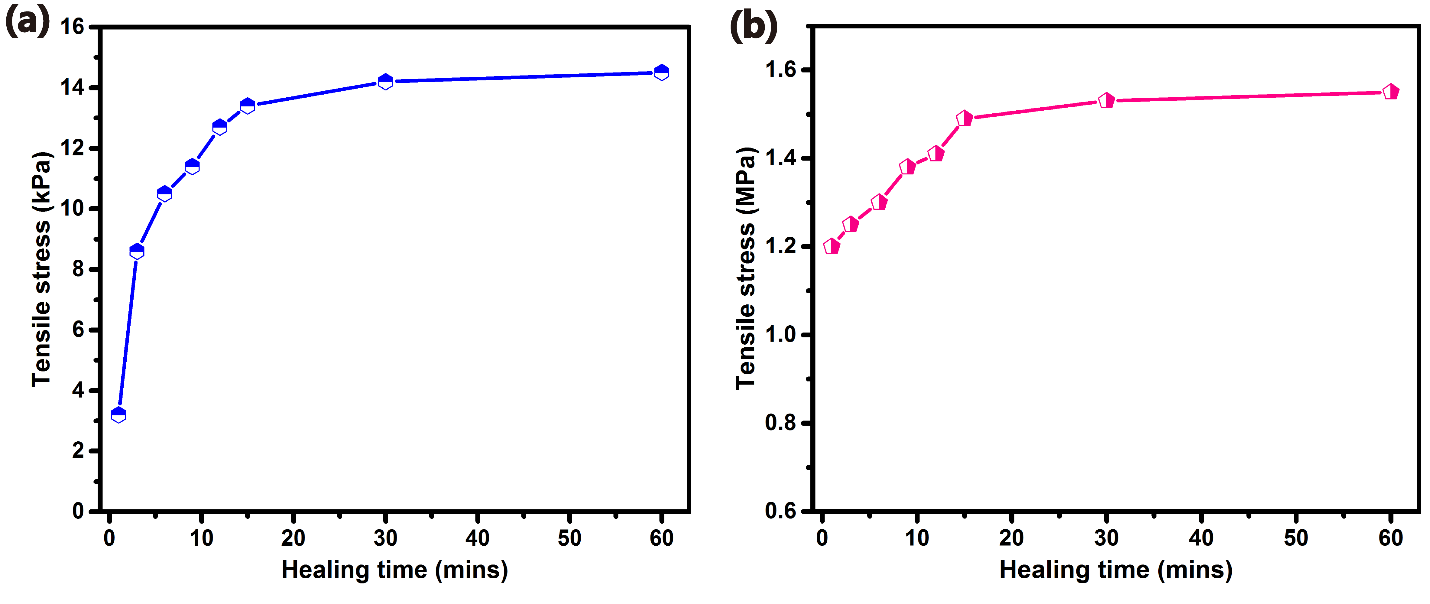


**Figure S15.** Tensile stresses of the PAA hydrogel layer and PU layer with different healing times when the strain reached 20%. It was found that mechanical strengths increased with increasing the healing time both for the electrode layer and EL layer, where the they almost stayed stable after 30 mins for healing.


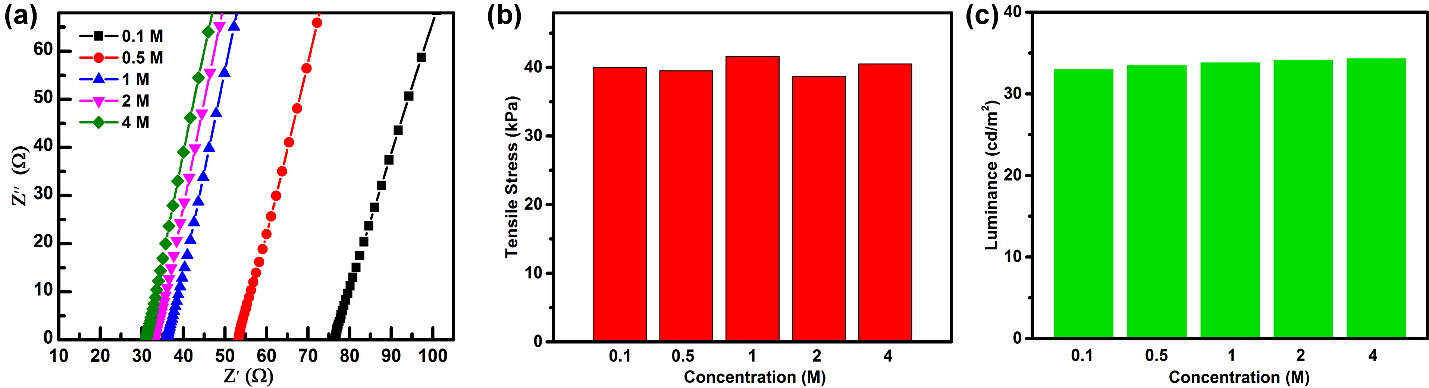


**Figure S16.** (a) The impedance spectra of PAA hydrogel of different NaCl concentrations. (b) The tensile stress of fracture points of PAA hydrogels with different NaCl concentrations showed slight variation, indicating the key parameter to determine the self-healing properties was the polymer framework of hydrogel. (c) The luminance intensity of EL devices based on PAA electrodes with different NaCl concentration at the driving voltage (2 V µm^-1^, 500 Hz), where the luminance increased slightly with higher NaCl concentration, ascribing to the capacitive behavior of phosphor layer when in working states and thus the variation of the electrode resistance would not affect the driving voltage distributed on the phosphor layer.


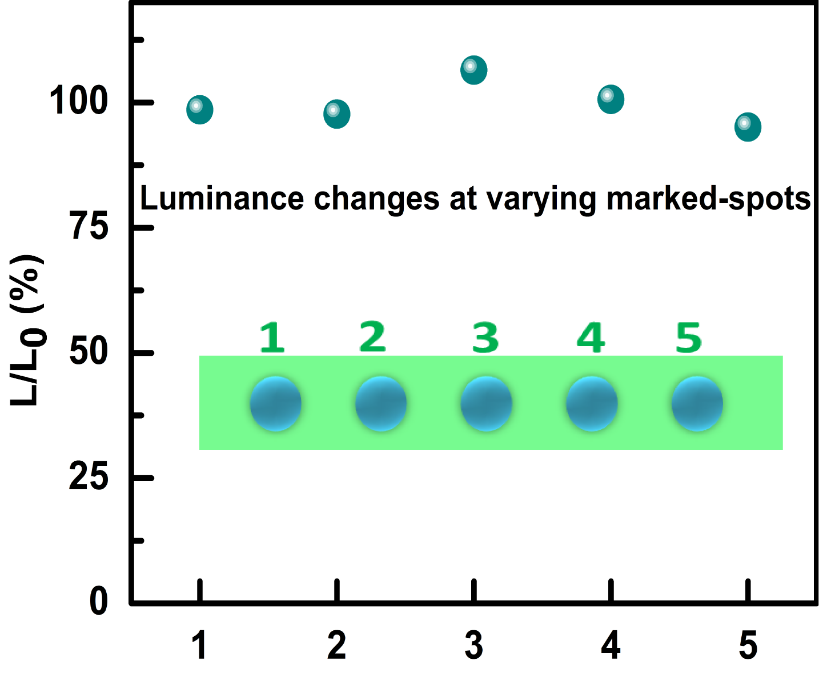


**Figure S17.** Luminance variation of different spots on the EL device after the healing process. L_0_ and L correspond to the luminance in the initial state and the healed state of 5 marked spots, respectively.


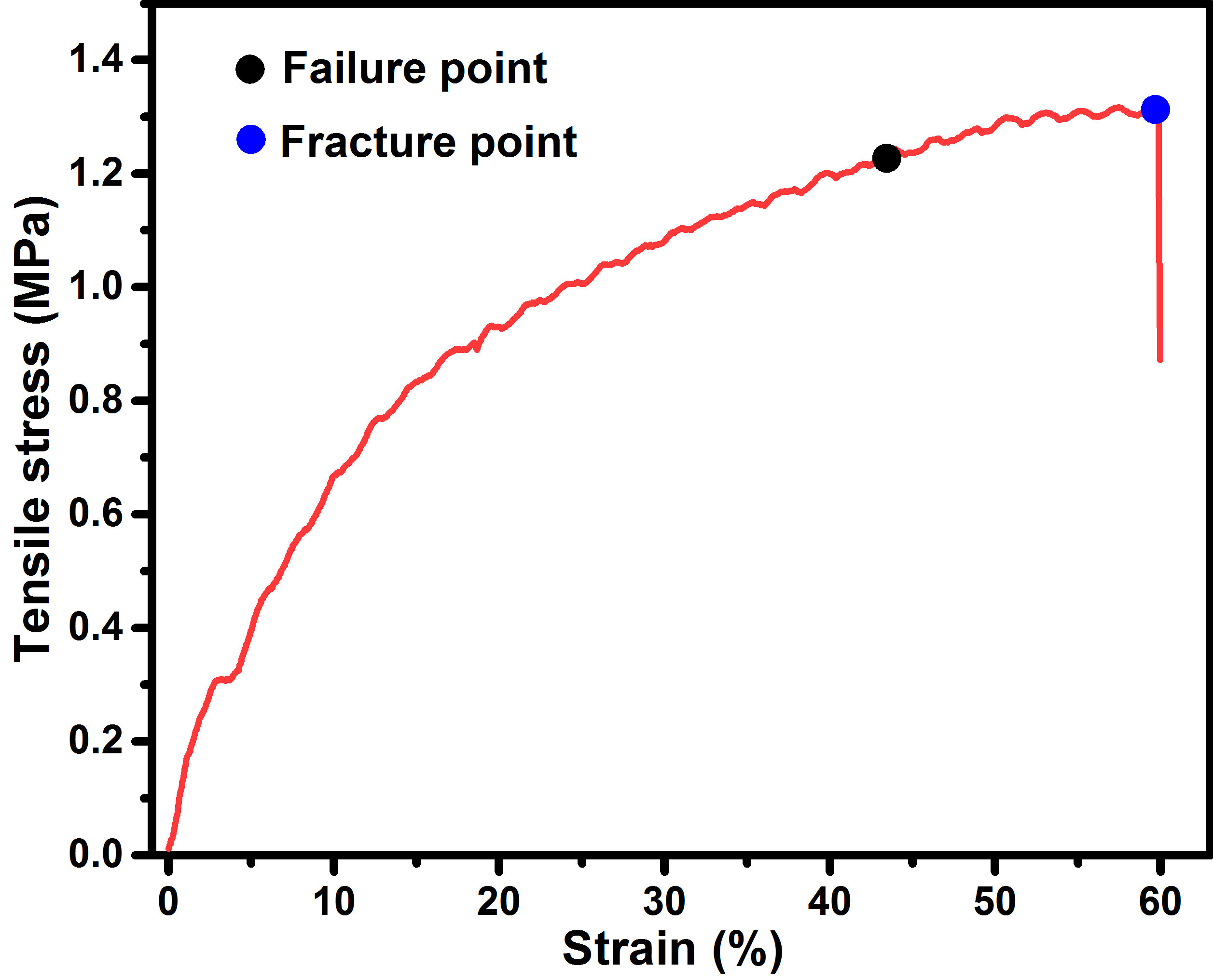


**Figure S18.** Tensile test of the EL device after first cut-healing process. It is revealed that the fracture point of the tensile stress was at 1.31 MPa with the strain at 60.2%, while the device could luminance up to the device failure point of 1.21 MPa with the strain at 41.6%. The failure point came before the fracture point, which might ascribe to the failure of healed electrode at healed region.


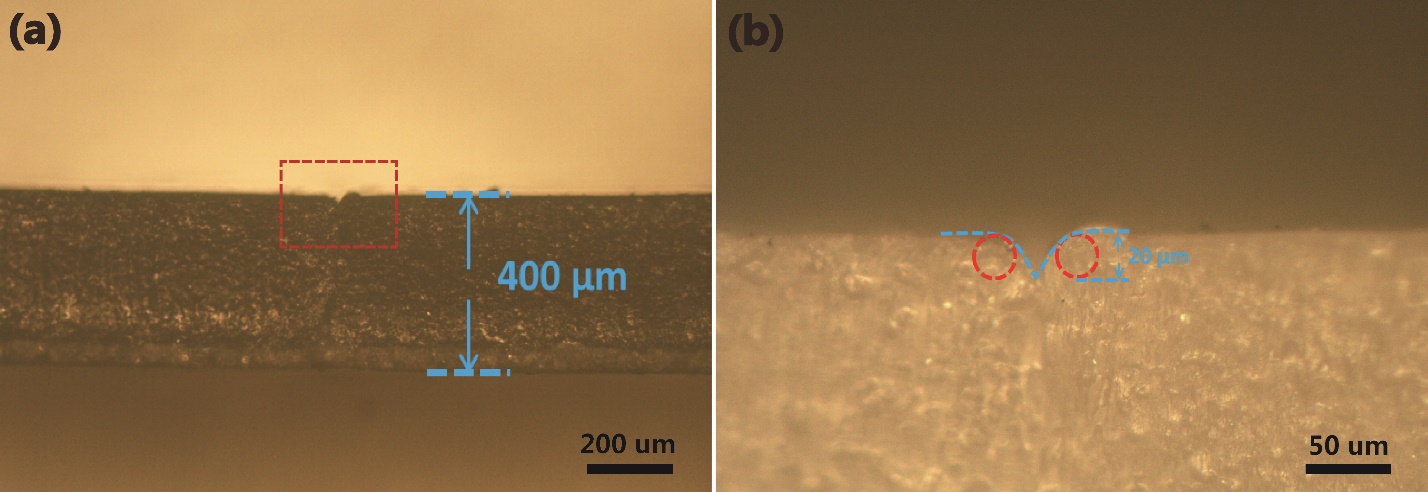


**Figure S19.** (a) Cross-section image of phosphor composite layer. (b) Magnified image of marked region in (a).


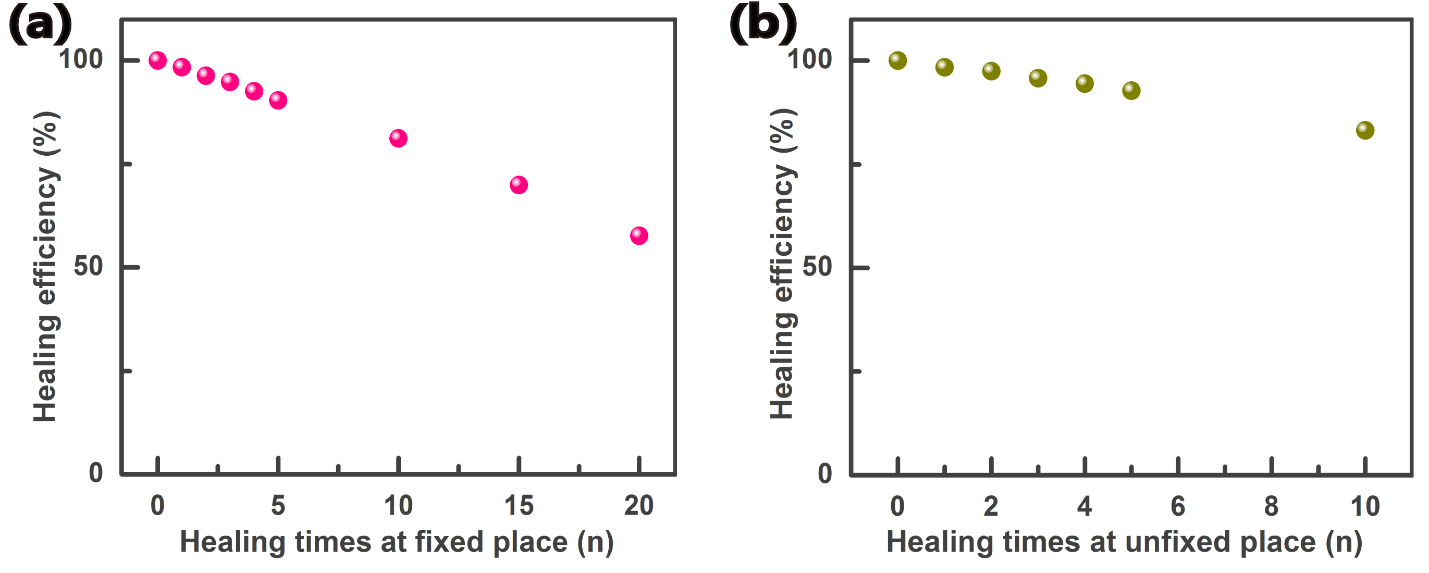


**Figure S20.** The healing efficiency of luminance with different healing times in fixed cut mode and unfixed cut mode, where 57.7% for 20 healing cycles at a fixed spot (a) and 83.2% for 10 healing cycles at unfixed spots (b).


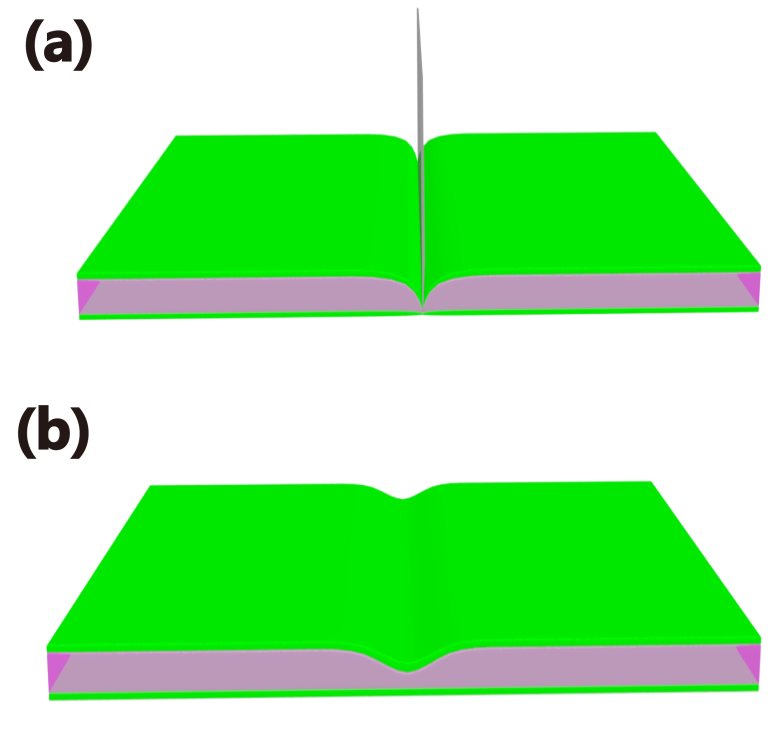


**Figure S21.** Schematic illustration of the irreversible shape deformation at cutting-healing region. (a)The shape deformation caused during cut. (b) The recovered device after cutting-healing process and the shape deformation region remains. It should be pointed out that the deformation size is just at micron level, which can be verified in SEM images (Figure 1d, Figure S15). This deformation at the healed region is believed to cause the redistribution of electric field and then the resulting changes of luminance.


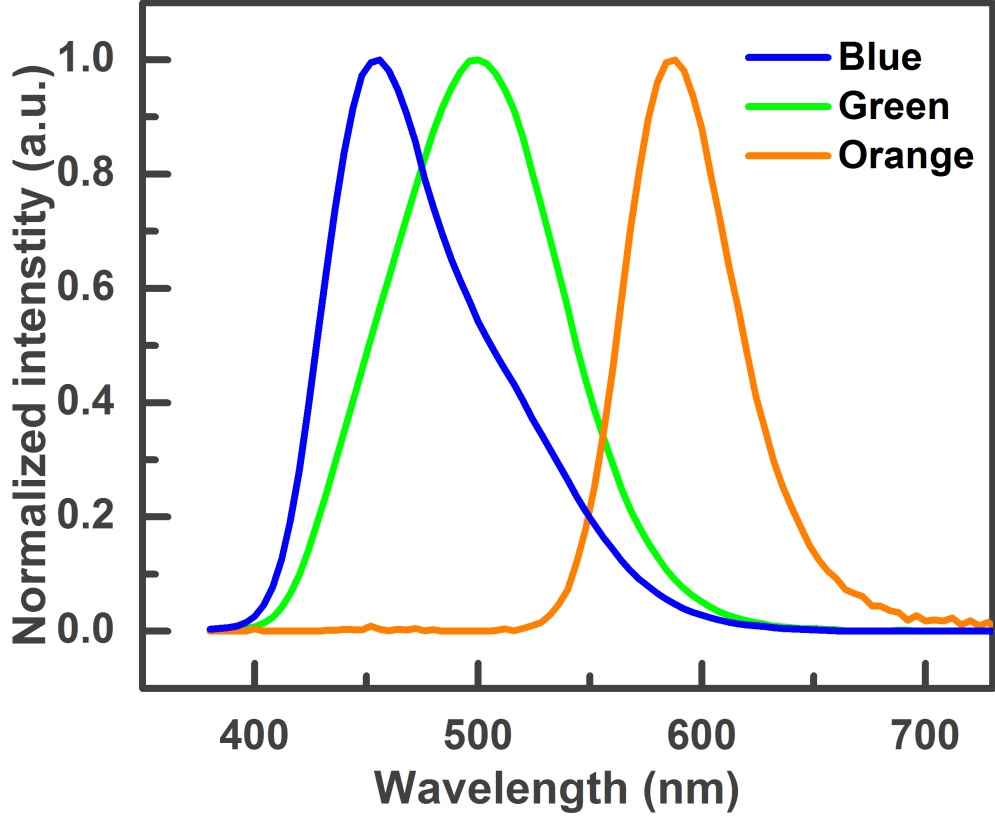


**Figure S22.** Emission spectra respectively corresponded to blue, green and orange colors of EL units for colorful LEGO assembled light-emitting devices.

**Movie S1**

A self-healing EL device goes through the initial, working state, cut, healing and revived working state.
